# A viral insulin-like peptide inhibits IGF-1 receptor phosphorylation and regulates IGF1R gene expression

**DOI:** 10.1016/j.molmet.2023.101863

**Published:** 2024-01-03

**Authors:** Martina Chrudinová, Nicholas S. Kirk, Aurelien Chuard, Hari Venugopal, Fa Zhang, Marta Lubos, Vasily Gelfanov, Terezie Páníková, Lenka Žáková, Julianne Cutone, Matthew Mojares, Richard DiMarchi, Jiří Jiráček, Emrah Altindis

**Affiliations:** 1 Boston College Biology Department, Chestnut Hill, MA, USA; 2 WEHI, Parkville, VIC, Australia; 3 Department of Medical Biology, Faculty of Medicine, Dentistry and Health Sciences, University of Melbourne, Parkville, VIC, Australia; 4 Ramaciotti Centre for Cryo-Electron Microscopy, Monash University, Clayton, VIC, Australia; 5 Department of Chemistry, Indiana University, Bloomington, IN, USA; 6 Institute of Organic Chemistry and Biochemistry, Czech Academy of Sciences, Prague, Czech Republic; 7 Novo Nordisk, Indianapolis, IN, USA

**Keywords:** Viral insulin/IGF-1 like peptides (VILPs), IGF-1, Insulin, IGF1 receptor, IGF1 receptor inhibition, Biased signaling, Iridoviridae

## Abstract

**Objective:**

The insulin/IGF superfamily is conserved across vertebrates and invertebrates. Our team has identified five viruses containing genes encoding viral insulin/IGF-1 like peptides (VILPs) closely resembling human insulin and IGF-1. This study aims to characterize the impact of Mandarin fish ranavirus (MFRV) and Lymphocystis disease virus-Sa (LCDV-Sa) VILPs on the insulin/IGF system for the first time.

**Methods:**

We chemically synthesized single chain (sc, IGF-1 like) and double chain (dc, insulin like) forms of MFRV and LCDV-Sa VILPs. Using cell lines overexpressing either human insulin receptor isoform A (IR-A), isoform B (IR-B) or IGF-1 receptor (IGF1R), and AML12 murine hepatocytes, we characterized receptor binding, insulin/IGF signaling. We further characterized the VILPs’ effects of proliferation and IGF1R and IR gene expression, and compared them to native ligands. Additionally, we performed insulin tolerance test in CB57BL/6 J mice to examine in vivo effects of VILPs on blood glucose levels. Finally, we employed cryo-electron microscopy (cryoEM) to analyze the structure of scMFRV-VILP in complex with the IGF1R ectodomain.

**Results:**

VILPs can bind to human IR and IGF1R, stimulate receptor autophosphorylation and downstream signaling pathways. Notably, scMFRV-VILP exhibited a particularly strong affinity for IGF1R, with a mere 10-fold decrease compared to human IGF-1. At high concentrations, scMFRV-VILP selectively reduced IGF-1 stimulated IGF1R autophosphorylation and Erk phosphorylation (Ras/MAPK pathway), while leaving Akt phosphorylation (PI3K/Akt pathway) unaffected, indicating a potential biased inhibitory function. Prolonged exposure to MFRV-VILP led to a significant decrease in IGF1R gene expression in IGF1R overexpressing cells and AML12 hepatocytes. Furthermore, insulin tolerance test revealed scMFRV-VILP's sustained glucose-lowering effect compared to insulin and IGF-1. Finally, cryo-EM analysis revealed that scMFRV-VILP engages with IGF1R in a manner closely resembling IGF-1 binding, resulting in a highly analogous structure.

**Conclusions:**

This study introduces MFRV and LCDV-Sa VILPs as novel members of the insulin/IGF superfamily. Particularly, scMFRV-VILP exhibits a biased inhibitory effect on IGF1R signaling at high concentrations, selectively inhibiting IGF-1 stimulated IGF1R autophosphorylation and Erk phosphorylation, without affecting Akt phosphorylation. In addition, MFRV-VILP specifically regulates IGF-1R gene expression and IGF1R protein levels without affecting IR. CryoEM analysis confirms that scMFRV-VILP’ binding to IGF1R is mirroring the interaction pattern observed with IGF-1. These findings offer valuable insights into IGF1R action and inhibition, suggesting potential applications in development of IGF1R specific inhibitors and advancing long-lasting insulins.

## Highlights

• MFRV and LCDCV-Sa viral insulins (VILPs) effectively bind to human IR and IGF1R and stimulate downstream signaling pathways.
• scMFRV-VILP selectively inhibits IGF1R autophosphorylation and Erk phosphorylation, while leaving Akt phosphorylation unaffected.
• scMFRV-VILP leads to downregulation of IGF1R gene expression, offering a novel approach for modulation of IGF1R levels.
• scMFRV-VILP exhibits a prolonged glucose-lowering effect in mice, showcasing its potential as a long-lasting insulin analog.
• CryoEM analysis demonstrates a highly analogous structure between scMFRV-VILP and IGF-1 when bound to IGF1R.

## Abbrevations

VILP: viral insulin/IGF like peptide
MFRV: mandarin fish ranavirus
LCDV-Sa: lymphocystis disease virus
dc: double chain
sc: single chain
IGF: insulin like growth factor
IGF-1: insulin like growth factor-1
IGF-2: insulin like growth factor-2
IGF1R: insulin like growth factor-1 receptor
IR: insulin receptor
ITT: insulin tolerance test
cryoEM: cryogenic electron microscopy
IRS: insulin receptor substrate

## 1 Introduction

The insulin/IGF system regulates cell metabolism, proliferation, and differentiation in vertebrates. It consists of three peptide hormones: insulin, IGF-1, and IGF-2, as well as their membrane receptors, including two isoforms of insulin receptor (IR-A and IR-B) and IGF-1 receptor (IGF1R), all belonging to the receptor tyrosine kinase (RTK) family [1,2]. While insulin and IGF-1 have the highest affinity for their cognate receptors, significant structural homology between the hormones and the receptors allows all hormones to bind and stimulate all receptors, albeit with different potencies. IGF-2 does not have a cognate receptor, but it can bind and activate IR-A and IGF1R with relatively high affinity [3,4].

**Figure 1:**
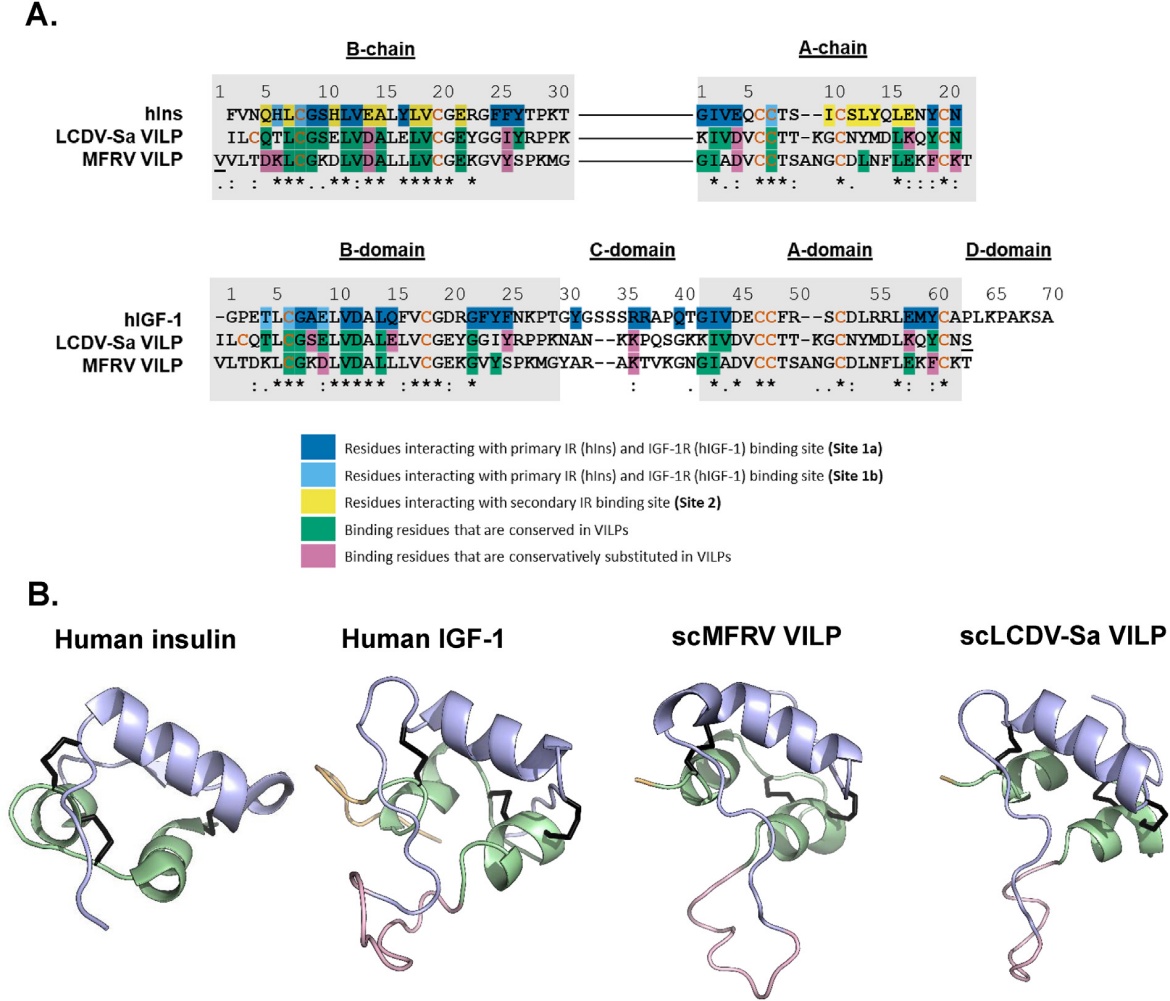
**Comparison of primary and predicted structures of MFRV and LCDV-Sa-VILPs with human insulin and IGF-1. A.** Sequence alignment of VILPs (as synthesized) with human insulin and IGF-1. The sequence of double chain (dc) VILPs and their comparison to human insulin is shown in the upper panel and the sequence of single chain (sc) VILPs and their comparison to human IGF-1 is shown in the lower panel. The underlined residues represent substitutions compared to the human ligands or differences between the sc and dc forms of the VILPs as synthesized. Note that in the natural sequence, Ala3 in LCDV-Sa is replaced with Cys. The residues important for receptor binding are highlighted as indicated in the figure legend. Cysteine residues are in red. **B:** 3D structures of human insulin (PDB:1MSO) and IGF-1 (PDB: 2GF1) and predicted 3D structure of scVILPs. A-chains/domains are in green, B-chains/domains are in blue, C-domains are in pink and D-domains are in orange. (For interpretation of the references to color in this figure legend, the reader is referred to the Web version of this article.)

Vertebrate insulin is a double chain peptide, comprising an A-chain and a B-chain (Figure 1A) linked by two interchain disulfide bonds and an additional intrachain disulfide bond within the A-chain [5]. IGFs are single chain peptides that share structural similarities with insulin, consisting of A- and B-domains corresponding to the A- and B-chains of insulin. However, IGFs also possess a C-domain inserted between the B- and A-domain, as well as an additional D-domain, extending from the C-terminus of the A-domain. IGFs contain analogous disulfide bonds to those found in insulin [6,7]. Insulin-like peptides (ILPs) are also present in invertebrates. ILPs play crucial roles in regulating metabolism, growth, development, life span, longevity, reproduction, and stress responses. While there are few exceptions, they typically bind to a single receptor [[8], [9], [10]].

Despite tremendous efforts to understand this complex system, the presence of *viral insulin/IGF-like peptides* (*VILPs*) and their effects on mammalian cells remained unknown until our recent discovery [11]. We identified five viruses belonging to *Iridoviridae* family possessing VILPs with ∼30–50 % sequence homology to human insulin and IGF-1 [11,12]. We discovered that VILPs can induce atypical effects on the insulin/IGF system, thereby offering new insights into these critical signaling systems.

In our previous studies, we focused on the VILPs identified in Lymphocystis disease virus-1 (LCDV-1), Grouper iridovirus (GIV) and Singapore grouper iridovirus (SGIV). We chemically synthesized both single chain [11] (sc, i.e. IGF-like, including the C-peptide region) and double chain [12,13] (dc, i.e. insulin like, without the C-peptide region) forms of each VILP and characterized their effects on the insulin/IGF system. We showed that all VILPs could bind and activate IR and IGF1R. Both forms of SGIV and GIV VILPs stimulated downstream signaling, cell proliferation and lowered blood glucose in mice [11,12]. In addition, in vivo infusion experiments revealed that dcGIV-VILP had a relatively stronger effect on white adipose tissue glucose uptake and gene expression than insulin, indicating specific effects on this tissue [12]. Conversely, we showed that scLCDV-1-VILP is a natural competitive antagonist of IGF1R [13,14]. Therefore, VILPs exhibit unique properties differentiating them from the native ligands, likely acquired through distinct co-evolution in the viral genomes to facilitate viral pathogenesis in the host [15,16].

This study focused on characterization of two novel VILPs identified in Mandarin fish ranavirus (MFRV) and Lymphocystis disease virus-Sa (LCDV-Sa). We chemically synthesized the VILPs in both sc and dc forms, and demonstrated their effective binding to human IR and IGF1R. ScMFRV-VILP inhibited IGF1R phosphorylation at high concentrations, downregulated IGF1R gene and protein expression, and had prolonged glucose-lowering effects in mice. Cryo-EM analysis revealed that scMFRV-VILP interacts with IGF1R receptor similarly to IGF-1. Our findings add to the evidence that VILPs possess unique properties distinct from the native hormones. These findings enhance our understanding of insulin/IGF signaling mechanisms and have the potential to facilitate the development of insulin/IGF analogs for clinical use.

## 2 Materials and methods

### 2.1 Bioinformatics

The sequence alignments presented in this paper were prepared using a multiple sequence alignment program (Clustal Omega). The sequence alignments in this paper were generated using Clustal Omega, a multiple sequence alignment program. It's important to note that while MFRV-VILP and IGF-1 share two residues in the C-domain (equivalent to Gly30 and Tyr31 of IGF-1), these specific residues were not directly aligned by the Clustal Omega software used for our sequence alignments in Figure 1A. Structural modeling of the VILPs were completed using I-TASSER [17]. The final figures were prepared using PyMOL. SignalP 6.0 was used for signal peptide prediction [18].

### 2.2 Peptide synthesis of scVILPs

The single-chain VILPs were assembled on 0.1 mmol Rink amide ChemMatrix® resin using an ABI-433 A peptide synthesizer and Fmoc/6-Cl-HOBt/DIC coupling protocols. Fmoc-Asp-OtBu was employed to introduce the C-terminal Asn. Cleavage was conducted by treatment with 10 mL of TFA solution containing 2.5 % TIS, 2.5 % 2-mercaptoethanol, 2.5 % anisole, and 2.5 % H2O at room temperature with gentle agitation for 1.5 h. The resin was filtered, and the peptide precipitated by addition of cold ether (50 mL). The peptide precipitate was collected by centrifugation then washed with cold ether (3 × 50 ml). The crude peptide was solubilized in mixture of 0.1 M ammonium acetate, 6 M urea (15 mL) and acetic acid (20 mL). I2 in methanol was added dropwise, and the solution was further stirred for 20 min before quenching with ascorbic acid. The intermediate with two disulfide bonds was obtained after preparative HPLC purification and lyophilization. The Met residue was oxidized to sulfoxide in this step. The lyophilized peptide was dissolved in TFA with the addition of DMSO (5 %). The mixture was stirred at room temperature for 2 h. After cold ether precipitation, the peptide was solubilized in 20 % CH3CN/H2O and adjusted to pH 8.0. The solution was acidified after 30 min at room temperature. Another preparative HPLC purification and lyophilization afforded the final product. HPLC purifications were performed on a Waters instrument (Waters Controller model 600, Waters dual wavelength detector 2487, ProStar model 701 fraction collector and Kipp & Zonen BD41 chart recorder). Luna 10μ C8 100 A AXIA (250 × 21.2 mm) column with flow rate of 12 mL/min was used. Data was collected using uv-visible absorption at 220 nm. Analytical HPLC was performed on an Agilent 1260 Infinity with Phenomenex Kinetex C8 2.6μ 100 A (75 × 4.6 mm) column. Flow rate of 1 mL/min and a gradient of 10 %–80 % acetonitrile in water with 0.1 % of trifluoroacetic acid over 10 min (with additional 5 min wash and pre-equilibration) were used for most of the analyses. Data was collected using uv-visible absorption at 214 nm. The original sequence of VILP identified in LCDV-Sa viral genome has a Cys in position 3. However, due to expected complications with protein folding, we substituted Cys3 with Ala. The final sequence of the VILPs synthesized are shown in Figure 1A. As indicated in the figure**,** Ser22 in LCDV-Sa is only present in scLCDV-Sa but not in dcLCDV-Sa. Val in position −1 in MFRV VILP is only present in dcMFRV but not scMFRV. The LC-MS spectra of both sc VILPs are shown in Figure S1.

### 2.3 Peptide synthesis of dcVILPs

DcLCDV-Sa VILP was synthesized according to Kosinova et al. [19]. The A-chain and the B-chain were synthesized by stepwise coupling of the corresponding Fmoc amino acid on Fmoc-Asn(Trt)-Wang LL resin and Fmoc-Lys (Boc)-Wang LL resin, respectively, using an automatic solid-phase synthesizer on the Spyder Mark IV Multiple Peptide Synthesizer (EP 17206537.7), developed in the Development Center of the Institute of Organic Chemistry and Biochemistry (http://dc.uochb.cz/index.php). HBTU/HOBt in DMF and DIC/HOBt/DIPEA were used as coupling reagents. Fully protected peptides were cleaved from the resins by a TFA/H_2_O/TIS/EDT/phenol/thioanisol mixture (90:3:1:1:2:3) and were precipitated with cold diethyl ether. Crude A- or B-chains (100 μmol) in reduced (SH) forms were dissolved and stirred in 25 mL of freshly prepared sulfiltolysis buffer (100 mM Tris, 250 mM Na_2_SO_3_, 80 mM Na_2_S_4_O_6_, and 7 M GuaHCl pH 8.6) for 3 h at room temperature to convert SH groups to S-sulfonates. The chains were then desalted on a Sephadex G10 column (4 cm × 85 cm) in 50 mM NH_4_HCO_3_ and purified using reversed-phase high-performance liquid chromatography (RP-HPLC) (Nucleosil C18 column, 250 mm × 21 mm, 5 μm). Next, the A-chain (27 mg) and the B-chain (34 mg) S-sulfonate derivatives were dissolved in 1.7 mL and 2.1 mL of degassed 0.1 M Gly/NaOH buffer (pH 10.5), respectively. The exact molar concentrations of individual chains were determined by UV spectrophotometry at 280 nm using molar extinction coefficients of 3040 and 2800 M^−1^ cm^−1^ for the A-chain and the B-chain, respectively. The solutions of chains were combined, and dithiothreitol (DTT, aliquoted from Pierce, catalog no. 20291) in a minimal volume of a degassed 0.1 M Gly/NaOH buffer (pH 10.5) was rapidly added to the peptide solution to give a SH:SSO_3_^−^ molar ratio of 1.2. This solution was stirred for 120 min in a capped vessel at room temperature. After the reduction of SSO_3_^−^ to SH, 3.8 mL of aerated 0.1 M Gly/NaOH buffer (pH 10.5) was added, and the resulting solution was stirred for 48 h at 4 °C in an open vessel to permit air oxidation. Glacial acetic acid (3.8 mL) was added to the mixture to terminate the reaction. The resulting mixture was applied to a low-pressure column (Sephadex G-50 in 1 M acetic acid, 2 cm × 75 cm). The fraction containing the dcLCDV-Sa-VILP was purified using RP-HPLC (Nucleosil C18 column, 250 mm × 8 mm, 5 μm). The molecular weight of the analogue was confirmed by HR mass spectroscopy (LTQ Orbitrap XL, Thermo Fisher Scientific, Waltham, MA) and the purity of analogue was controlled by RP-HPLC (Nucleosil C18 column, 250 mm × 4 mm, 5 μm) at 1 mL/min using the following gradient of acetonitrile in water with 0.1 %: TFA (v/v): 0 min 8 % ACN, 1 min 28 % ACN, 21 min 36 % ACN, 34 min 44 % ACN, 36 min 72 % ACN, 37 min, 8 % ACN and monitored at 218 and 254 nm (Fig. S2A). The correct molecular weight of dcLCDV-Sa-VILP was confirmed by HR mass spectroscopy (LTQ Orbitrap XL, Thermo Fisher Scientific, Waltham, MA) (Fig. S2B).

DcMFRV-VILP was synthesized according to Liu et al. [20]. The A-chain and the B-chain were synthesized by stepwise coupling of the corresponding Fmoc amino acid using an automatic solid-phase synthesizer mentioned above. Synthesis of A-chain was performed on Fmoc-Thr (tBu)-Wang LL resin (0.06 mmol scale). The resin-bound A-chain was treated with 25 % β-mercaptoethanol in DMF (v/v, 6 mL) for 1.5 h at rt. The reaction was repeated once more and a test cleavage with MS analysis was performed. The resulting resin was washed with DMF (3 × 4 mL) and DCM (3 × 4 mL) and 2,2′-dithiobis (5-nitropyridine) (DTNP, 10 eq) in DCM (4 mL) was added, reaction was carried out for 1 h at rt. The resin-bound peptide was washed with DMF and DCM as mentioned above and treated with 1 % TFA, 5 % TIS in DCM (4 mL), 5 × 2 min. The resin was washed again with DMF and DCM and agitated in DCM (4 mL) for 1 h at rt. A-chain was cleaved from resin by treatment with TFA:TIS:H_2_O (95:2.5:2.5, v/v/v) for 1.5 h at RT. The peptide was then precipitated by cold diethyl ether, pellet was collected by centrifugation, washed twice with cold ether, dried, dissolved in a mixture of ACN and H_2_O and lyophilized. Synthesis of the B-chain was performed on Fmoc-Gly-Wang LL resin (0.066 mmol scale). Cleavage was achieved by treatment with TFA:TIS:H_2_O (95:2.5:2.5, v/v/v) with DTNP (15 eq) for 2 h at RT. Peptide was collected as described for the A-chain. Both chains were purified by Waters HPLC system (Waters 600 with 2487 Dual λ Absorbance Detector), using a Nucleosil 100-7 C8 column (250 × 10 mm, 7 μm, Macherey–Nagel) at a flow rate of 4 mL/min and the following gradient: t = 0 min/10 % B, t = 30 min/100 % B, t = 31 min/10 % B. Solvent A is 0.1 % TFA in H_2_O and solvent B is 80 % ACN in A (v/v). Compounds were detected at 218 and 254 nm. A-chain (14 mg, 5.34 μmol) and B-chain (20 mg, 5.95 μmol) were mixed and dissolved in 6 M urea, 0.2 M NH_4_HCO_3_ buffer (pH 8, 2.5 mL). The mixture was stirred for additional 5 min at RT, solution of iodine (34 mg, 0.134 mmol, 25 equiv based on A-chain) in AcOH (9 mL) was added and the reaction was stirred for 10 min at RT. The reaction was stopped by the addition of 1 M ascorbic acid until the iodine color disappeared. The solution was diluted by H_2_O and purified as described above. The purity of both chains and final product (Fig. S3A) was checked by HPLC on a Watrex HPLC system (Watrex DeltaChrom™ P200 binary Pump and Wufeng LC-100 UV Detector), using a Nucleosil 120-5 C8 column (250 × 4.6 mm, 5 μm, Macherey–Nagel) at a flow rate of 1 mL/min, with the same gradient and solvents as described for the preparative HPLC. The correct molecular weight of dcMFRV-VILP was confirmed by HR mass spectroscopy (LTQ Orbitrap XL, Thermo Fisher Scientific, Waltham, MA) (Fig. S3B).

### 2.4 Cell culture

Human IM-9 lymphocytes (LGC Standards Sp. z.o.o., in partnership with ATCC, #CCL-159, Poland) and murine embryonic fibroblasts, that were derived from IGF1R knockout mice [21] and stably transfected with either IR-A (R^−^/IR-A cells), IR-B (R^−^/IR-B cells) or IGF1R (R^+^ cells) [22], kindly provided by A. Belfiore (Catanzarro, Italy) and R. Baserga (Philadelphia, PA), were cultured as described previously [23,24]. AML12 (ATCC, #CRL-2254, USA) cells were cultured in DMEM/F12 50/50 medium (Corning) supplemented with with 10 % FBS (Fisher Scientific), 100U/ml penicillin and 100 μg/ul streptomycin (Gibco). HEK293 cells were cultured in high glucose DMEM (Cytiva) supplemented with 100 IU/mL penicillin, 100 μg/mL streptomycin, 10 mM Hepes, and 0.25 % bovine growth serum (HyClone SH30541). All cell lines were culcured at at 37 °C in a humidified atmosphere containing 5 % CO_2_.

### 2.5 Binding competition assay

For receptor binding studies, human IM-9 lymphoblasts, that express IR-A exclusively, and R^−^/IR-B and R^+^ murine embryonic fibroblasts were used for a whole-cell receptor-binding assay. Receptor binding assays on IR-A were perfomed accoding to Morcavallo et al. [24]and binding assays on IR-B and IGF1R were performed according to Kosinova et al. [19]. The binding curve of each ligand was determined in duplicate, and the final dissociation constant (K_d_) was calculated from at least three (n ≥ 3) binding curves. Human insulin and human IGF-1 were supplied by Merck. Human ^125^I-insulin was prepared as described by Asai et al. [25] and human ^125^I-IGF-1 was prepared as described in Kertisova et al. [26].

### 2.6 Receptor phosphorylation assay

Insulin or IGF-1 receptor phosphorylation was measured as described in [13]. Briefly, HEK293 cells overexpressing human IR-A, IR-B, or IGF-1R were plated in 96-well tissue culture plates and cultured for 16–20 h at 37 °C, 5 % CO2, and 90 % humidity. Serial dilutions of biosynthetic human insulin, IGF-1 and test peptides were prepared in DMEM supplemented with 0.5 % BSA and were added to the plate wells. For antagonism studies, test peptides were pre-mixed with 10 nM IGF-1. After 15 min incubation at 37 °C in humidified atmosphere with 5 % CO2, the cells were fixed with 5 % paraformaldehyde for 20 min at room temperature, washed twice with PBS (pH 7.4) and blocked with 2 % BSA in PBS for 1 h. The plate was washed three times, filled with anti-phospho-IR/IGF1R (Tyr1158) antibody (Millipore) and incubated for 3 h at room temperature, after which the plate was washed four times and filled with goat anti-rabbit secondary antibody (#A16110, ThermoFisher). Following 30 min incubation and another four washes, 0.1 mL of TMB One Solution substrate (#00-2023; Invitrogen) was added to each well. Color development was stopped 10 min later by adding 0.05 mL 1 M HCl. Absorbance at 450 nm was measured on Envision multimode reader (Perkin–Elmer). Absorbance vs. Peptide concentration dose–response curves were plotted, and EC_50_ or IC_50_ values were determined using logistic nonlinear three-parameter regression in GraphPad Prism 8 (GraphPad Software). Each experiment was performed at least three times and the error bars represent standard deviation.

### 2.7 Insulin/IGF signaling via IR-A, IR-B and IGF1R

For receptor phosphorylation and downstream signaling experiments, R^−^/IR-A, R^−^/IR-B and R^+^ cells (described in **2.4**) were used to explore signaling properties of ligands via specific receptors. Cells were seeded into 24-well plates (10^5^ cells per well) in 300 μl of culture medium (described above) and grown overnight. Afterwards, cells were washed twice with PBS and starved in serum-free culture medium for 4 h. After the starvation, cells were washed with pure DMEM medium and incubated with ligand diluted in pure DMEM medium (0, 0.1, 1, 10, 100, 250 and 500 nM) in 37 °C for 30 min. For co-incubation of a VILP with 10 nM insulin/IGF-1, increasing concentrations of the VILP were pre-mixed with insulin or IGF-1 prior stimulation of cells. The reaction was terminated by washing the cells with ice-cold PBS (HyClone) followed by snap freezing in liquid nitrogene. Cell lysis was performed using 50 μl of RIPA buffer (Millipore) supplemented with protease and phosphatase inhibitors (Bimake). Cells on plates were incubated in RIPA buffer on ice for 15 min, subsequently sonicated for 1 min in a bath sonicator and then transferred to microtubes. The lysates were centrifuged (13000 g, 5 min, 4 °C) and supernatant was transferred to new microtubes. Protein concentration in each sample was evaluated using BCA Assay (Thermo Fisher Scientific). Samples were further diluted using sample buffer for SDS-PAGE (final concentration 62.5 mM Tris, 2 % SDS (w/v), 10 % glycerol (v/v). 0.01 % bromphenol blue (w/v), 0.1 M DTT (w/v), pH 6.8 (HCl)) and routinely analyzed using SDS-PAGE and immunoblotting. Cell lysates (4 μg of protein content/sample) were separated on 10 % polyacrylamide gels and electroblotted to PVDF membrane (0.45 μm, Millipore). The membranes were probed with primary antibodies against phospho-IR/IGF1R (1:500, #3024), phospho-Akt (S473) (1:1000, #9271), phospho-Erk1/2 (T202/Y204) (1:5000, #9101), IRβ (1:1000, #3025), IGF1Rβ (1:1000, #9750), Akt (1:1000, #4685) and Erk1/2 (1:2000, #9102). All primary antibodies were purchased from Cell Signaling Technology. HRP Goat Anti-Rabbit secondary antibody was used in all cases (1: 10000, ABclonal #AS014).The western blots were developed using SuperSignal West Pico PLUS Sensitivity substrate (Thermo Fisher Scientific) on a Blue Autoradiography & Western Blotting Film (USA Scientific). Each experiment was repeated at least three times (n ≥ 3). Western blots were quantified using Image Lab 6.0.1 (Bio-Rad). Statistical analysis of each data point compared to 10 nM insulin/IGF-1 was performed using unpaired two-tailed Student's t-test.

### 2.8 Vector preparation and purification

The plasmids utilized in this study were generated by inserting synthesized MFRV-VILP, LCDV-Sa-VILP, or human IGF-1 genes between the NheI and HindIII restriction sites of the pcDNA3.1+ vector. Subsequently, these constructs were transformed into electrocompetent *E. coli*. To ensure the correct insertion and reading frame, colony PCR was performed followed by Sanger sequencing. Plasmids were then amplified using ZymoPURE II Plasmid MidiPrep Kit (Zymo Research, #D4201). The primers used for amplification of ligand constructs are listed in Table S1.

### 2.9 Signaling in transfected cells

R^+^ cells (described in **2.4**) were seeded into 24-well plate in a density 10^5^ cells per well in 300 μl of culture medium and grown overnight. Cells were transfected in Opti-MEM medium (Gibco) with respective plasmid (empty vector, MFRV-VILP, IGF-1 or LCDV-Sa-VILP vector), using 500 ng of vector per well and Lipofectamine 3000 according to manufacturer's instructions. Three hours post transfection, Opti-MEM medium was exchanged for regular culture medium. 11 h post transfection, cells were washed once with PBS and starved in serum free culture medium. Cells were grown for additional 24 h. For the final 30 min of incubation, IGF-1 diluted in serum free medium was added to chosen wells so that the final concentration was 10 nM per well. Serum free medium only was added to the non-stimulated wells. The reaction was terminated by washing the cells with ice-cold PBS followed by snap freezing in liquid nitrogene.

To determine the secretion of VILPs following transfection, AML12 cells were seeded into a 24-well plate in a density 15 × 10^4^ cells per well and grown overnight. Cells were then transfected with either an empty vector or an MFRV-VILP vector for 35 h as described above. Supernatants from these cells were collected upon completion of the experiment. Simultaneously, a plate seeded earlier with 15 × 10^4^ AML12 cells per well underwent two washes with PBS and was subjected to serum starvation for 4 h before stimulation. The supernatants collected from transfected cells were used to stimulate these serum-starved AML12 cells. Stimulation was conducted for 15 min at 37 °C, followed by snap-freezing and storage at −80 °C for subsequent analysis.

In all experiments, cell lysis and western blot analyses were performed as outlined in section 2.7. Statistical analysis was performed using ordinary one-way ANOVA followed by Tukey's multiple comparison test.

### 2.10 RNA isolation and RT-qPCR

R^+^ cells were cultured and transfected as described in **2.9**, including starvation of cells 11 h post-transfection. AML12 cells were seeded into a 24-well in a density of 10^5^ cells per well in 300 μl of culture medium (described in **2.4**) and grown overnight. Subsequently, they were tramsfected analogically to R^+^ cells. Samples of both cell lines were collected before transfection (basal) and 7, 11, 26 and 35 h post transfection. For lysis, cells were incubated for 5 min at RT in Tri Reagent (Molecular Research Center, Inc.).

To assess the potential impact of external VILP exposure on IGF1R gene expression, we employed R^+^ cells, seeding 30 × 10^4^ cells per well. Following a 24-hour incubation period, cells were washed twice with PBS and were subsequently serum-starved for 4 h before sequential stimulation with various ligands (scMFRV-VILP, dcMFRV-VILP, and IGF-1). The cells were stimulated with 100 nM of each ligand every 3 h over a 24-hour period. Post the final stimulation, the cells were snap-frozen and stored at −80 °C.

Subsequently, the lysates were transferred to microtubes and chloroform in ratio 5:1 (Tri Reagent: chloroform) was added. The mixture was vortexed for 15s, incubated at RT for 5 min and centrifuged for 15 min at 12000g, 4 °C. The upper aqueus phase was transferred to new tubes, 100 % ethanol was added to give a 1:1 volumetric ratio and subsequently the Direct-zol RNA Miniprep Kit (Zymo Research) was used according to the manufacturer's instructions. cDNA synthesis was performed using Maxima™ H Minus cDNA Syntheis Master Mix (ThermoFisher Scientific) according to manufacturer's instructions. The qPCR was performed using Power SYBR® Green PCR Master Mix (Applied Biosystems) according to manufacturer's instructions on QuantStudio 3 (Applied Biosystems). The primers used are listed in Table S2. Statistical analysis was performed using ordinary one-way ANOVA followed by Tukey's multiple comparison test.

### 2.11 Proliferation assay

R^+^ cells (described in 2.4) were seeded into 96-well plates so that they reached ∼50 % confluency after overnight incubation. On the second day, cells were transfected in Opti-MEM medium (Gibco) with respective plasmid (epmty vector, MFRV-VILP, or human IGF-1 vector) using 100 ng of vector per well and Lipofectamine 3000 according to manufacturer's instructions. Three hours post transfection, Opti-MEM medium was exchanged for regular culture medium. 24 h post transfection, cells were washed once with PBS and starved in serum free culture medium. After 24 h of starvation, cells were stimulated with IGF-1 (diluted in serum free medium) to reach final concentration of 10 nM IGF-1 per well for another 24 h period (serum free medium only was added to the non-stimulated wells). To determine the rate of proliferation in each well, BrdU Cell Proliferation Assay (Sigma–Aldrich, #QIA58) was used. Manufacturer's instructions were followed, with the BrdU probe being added for the last 6 h of the 24 h stimulation with IGF-1. Absorbance at dual wavelengths of 450–540 nm was measured using a spectrophotometric plate reader. The experiment was repeated at least three times and for each biological replicate, results were expressed as relative change compared to non-stimulated cells transfected with empty vector. Statistical analysis was performed using paired two-tailed Student's t-test.

### 2.12 Insulin tolerance test

All animal studies presented in this study complied with the regulations and ethics guidelines of the NIH and were approved by the Boston College Institutional Animal Care and Use Committee. Insulin tolerance testing was performed on 12 to 17-week-old male C57BL/6 J mice (Jackson Laboratory). Mice were grouped according to their weight before experiment. After 6-hour starvation, mice were injected i.p. with insulin (Humulin, 6 nmol/kg, corresponds to 1.0 U/kg, 1x) (Eli Lilly), scMFRV-VILP (6 nmol/kg, 1x and 60 nmol/kg, 10x), dcMFRV-VILP (60 nmol/kg, 10x), dcLCDV-Sa-VILP (0.3 μmol/kg, 50x), and saline as a control (n = 4–11 per condition). Tail-vein blood glucose was measured until 3 h post-injection using an Infinity glucometer (US Diagnostic Inc.). Statistical analysis was done using Mixed effects analysis - Dunnett's multiple comparisons test.

### 2.13 CryoEM analysis of scMFRV-VILP bound to IGF1Rzip

IGF1Rzip is a construct comprising a 30-residue signal peptide, followed by residues 1–905 of the intact IGF1 holoreceptor, a 33-residue leucine-zipper motif [27], a three-residue spacer, and an c-myc tag (11-residues). The construct was produced by stable expression and secretion from CHO–K1 cells and purified by 9E10 antibody-affinity chromatography and size-exclusion chromatography as previously described [28]. The final sample was prepared at a concentration of 1.4 mg mL^−1^ in 24.8 mM Tris–HCl (pH 8.0), 137 mM NaCl, 2.7 mM KCl plus 0.02 % NaN_3_ (“TBSA”).

ScMFRV-VILP was prepared from dry powder at a concentration of 147 μM in 10 mM HCl (1 mg mL^−1^). The resulting solution was combined 3/20 with IGF1Rzip sample prepared as above to provide a final sample concentration of 1.2 mg mL−1 IGF1Rzip (5.6 μM per monomer) and 22 μM MFRV-VILP in TBSA plus 2 mM HCl (∼4-fold molar excess of VILP calculated per receptor αβ monomer).

Quantifoil R1.2/1.3 300-mesh grids (Quantifoil Micro Tools GmbH; Germany) were glow discharged in a Pelco easiGlow device (Ted Pella; CA) at 15 mA for 30 s. 4 μL of the sample was applied to the grids, which were then blotted using a Vitrobot mark IV (ThermoFisher Scientific; operated at 4 °C and 100 % humidity, 3 s blot time, 0 s wait time, 0 blot-force) before being plunge frozen in liquid ethane.

CryoEM imaging was performed using a Titan Krios (ThermoFisher Scientific) equipped with a Gatan K3 camera, a Quantum-GIF energy filter, and data acquisition software EPU 2. Imaging was performed in nanoprobe energy-filtered zero loss mode using a 10 eV slit width. A nominal magnification of 130,000 × was used which provided a calibrated specimen level pixel size of 1.06 Å. A C2 condenser aperture of 50 μm was used and the K3 camera was operated in correlated double sampling mode at a dose rate of 11.2  e−.pixel−1 s−1. Datasets were collected using the aberration-free image shift (AFIS) method, with 21 movies collected per stage shift. Movies were collected using a 5.36 s exposure time fractionated into 60 sub-frames resulting in a total accumulated dose of 60 e^−^/Å^2^ per movie. Movies were collected at a defocus range of −0.4 μm to −1.7 μm. All movies were collected from a single grid (n = 1).

A combined dataset of 8108 movies was motion-corrected using the patch motion job in cryoSPARC 3.3 [29]. CTF parameter estimation was performed with the patch CTF job within cryoSPARC. 7833 movies were retained after removing 275 micrographs with poor motion trajectories, poor CTF fit or significant crystalline ice. 8.5 M particles were picked from the patch motion-corrected micrographs using the general model in crYOLO [30] and extracted, binned 4-fold, with a box size of 256 px. Binned particles were 2D classified into 200 classes within cryoSPARC and the classes with very poor class averages removed, leaving 5.2 M particles. 100 k random particles were used to generate 12 initial models with 0 class similarity. Two obvious ‘good’ models were retained – one with closed FnIII legs (“closed”) and one with open FnIII legs (“open”). A poor initial model from 1000 random particles that were excluded after 2D classification was also generated to remove bad particles. All good particles were then heterogeneously refined against the open and closed models along with two poor models, leaving 1.6 M particles in the “closed” class and 2 M particles in the “open” class. The retained particles were then heterogeneously refined against their corresponding initial model and one poor model to remove remaining poor particles. 1.4 M particles were retained in the “closed” class while 1.8 M particles were retained in the “open” class. The “open” class was not able to be processed further into a high resolution map due to extensive heterogeneity and orientation bias. The “closed” particles were extracted, binned to 1.272 Å.pixel^−1^ with a box size of 384 px and homogeneously refined against the initial model. The consensus-refined particles were then subjected to 3D classification without alignment into 10 classes, with the best class containing 201 k particles locally refined again. CTF refinement was then performed, with beam tilt and trefoil aberrations estimated first, followed by anisotropic magnification and finally per-particle defocus and per-micrograph astigmatism corrections before a further local refinement. 3D flexible refinement was then performed in cryoSPARC v 4.1 to give the final reconstruction [31]. The 3D flex model was trained with two latent dimensions on data cropped to 192 px and binned to 96 px, giving a Nyquist at training of 5 Å. The 3D flex model was used to perform flexible refinement giving the final map at 3 Å, determined by an independent FSC calculation within cryoSPARC. DeepEMhancer [32] was used to sharpen the map for atomic modelling.

Atomic coordinates from human IGF1R (PDB entry 5U8R) were extracted and docked manually into the cryoEM map using ChimeraX v 1.4 [33] and the interdomain regions adjusted by real-space refinement in COOT (0.9 EL within CCP4 v7.1) [34]. Real-space refinement restrained to the initial coordinates in PHENIX version 1.20.148 followed to generate restraints for further refinement [35]. A model of MFRV-VILP was generated using AlphaFold2 and manually docked into cryoEM density in COOT. Manual adjustments to improve density fit and Ramachandran statistics followed, along with a whole model relaxation in ISOLDE v1.4 [36]. Loops lying outside density were then removed, and improbable and poorly fit rotamers were adjusted manually in COOT. A final real space refinement in PHENIX followed. Statistics are available in Table S3.

## 3 Results

### 3.1 MFRV and LCDV-Sa-VILPs show significant homology in primary and predicted 3D structures with human insulin and IGF-1

To compare the primary structures of MVRV and LCDV-Sa-VILPs with human insulin and IGF-1, we conducted a comparative alignment analysis (Figure 1A). The analysis indicated that the primary sequences of both VILPs exhibit significant homology (∼50 %) in their A- and B-chains/domains with the native peptides, whereas the C-domains show notable differences (Table 1). Both peptides contain six cysteine residues that form intrachain and interchain disulfide bonds, which are essential for proper folding of insulin/IGF-like molecules. While LCDV-Sa-VILP shows slightly higher similarity to the B-chain of insulin and IGF-1 than MFRV-VILP, the reverse is true for the A-chain/domain (Table 1). The C-domains of the VILPs are short (10 residues) and resemble human IGF-1 C-domain (12 residues) more than insulin C-peptide (35 residues). Nonetheless, there is no conservation of residues between human insulin and MFRV-VILP C-domains, except for one residue (AlaC4) (Figure 1A, Table 1). In addition, the only conserved residues between IGF-1 and MFRV-VILP C-peptides are GlyC1 and TyrC2.

**Table 1:** Comparison of Conserved Residues among Human Insulin, Human IGF-1, and VILPs.

	B-chain/domain		C-peptide/domain		A-chain/domain	
	Insulin	IGF-1	Insulin	IGF-1	Insulin	IGF-1
LCDV-Sa	57 %	52 %	0 %	0 %	48 %	38 %
MFRV	47 %	45 %	3 %	0 %	52 %	52 %


	Site 1 binding residues		Site 2 binding resudues	
	Insulin	IGF-1	Insulin	IGF-1
LCDV-Sa	65 %	48 %	50 %	–
MFRV	41 %	43 %	58 %	–

The upper panel of the table shows the percentage of conserved amino acid residues that are shared between MFRV and LCDV-Sa-VILPs, human insulin, and human IGF-1. The lower panel of the table shows the percentage of amino acid residues that are known to be involved in receptor binding.

We compared the conservation of residues involved in the interaction between human insulin and IGF-1 with their respective receptors in LCDV-Sa-VILP and MFRV-VILP. Results show that LCDV-Sa-VILP has higher conservation of insulin Site1 residues (65 %) compared to MFRV-VILP (41 %). However, MFRV-VILP has higher conservation of insulin Site 2 residues (58 %) compared to LCDV-Sa-VILP (50 %). In terms of IGF-1 comparison, LCDV-Sa-VILP has slightly higher conservation in Site 1 binding residues (48 %) compared to MFRV-VILP (43 %) (Table 1).

To assess the similarity between the 3D structures of MFRV and LCDV-Sa-VILPs with insulin and IGF-1, we utilized I-TASSER [17] to develop scVILPs models. These models were then compared with the 3D structures of human insulin and IGF-1 (Figure 1B). Both scVILPs sequences could be easily threaded onto the canonical structure of human insulin and human IGF-1, including the two A-chain α-helices, the central B-chain α-helix, and the alignment of the three disulfide bonds.

### 3.2 MFRV and LCDV-Sa-VILPs bind to human IR-A, IR-B and IGF1R

**Figure 2:**
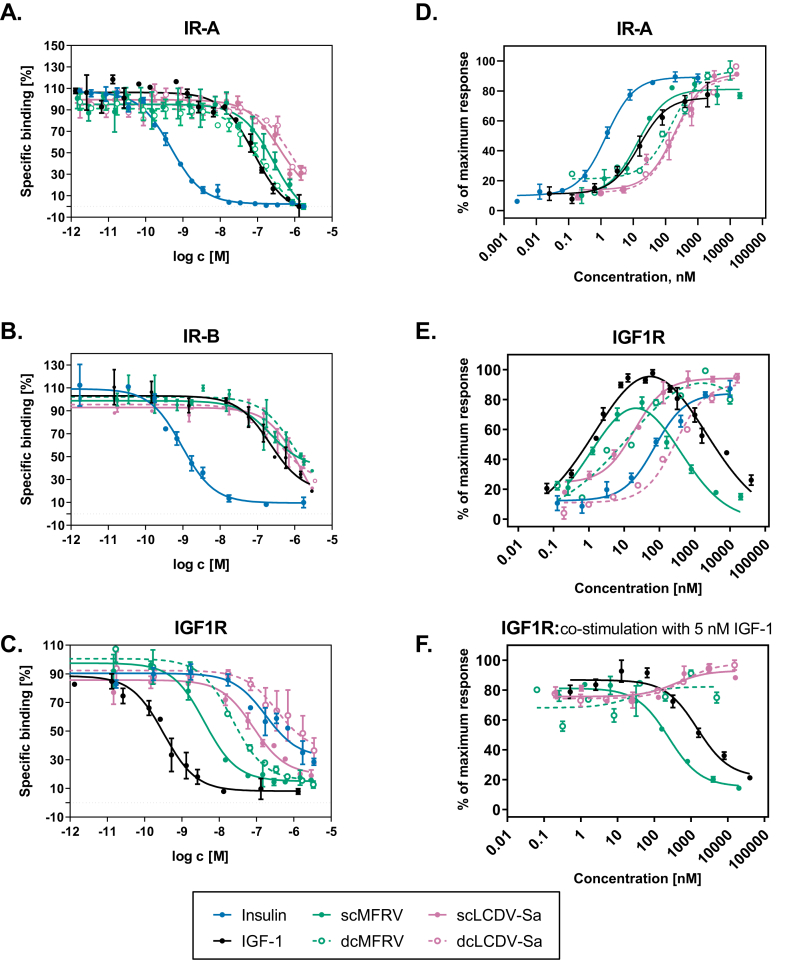
**MFRV and LCDV-Sa-VILPs can bind to human IR and IGF1R and stimulate autophoshorylation of the receptors. A-C**: Binding competition dose–response curves showing the ability of VILPs to compete with 125-I labeled human insulin for binding to IR-A **(A)** and IR-B **(B)**, and with 125-I labeled human IGF-1 for binding to IGF1R **(C)**. The experiments were performed using IM-9 cells for measurements on IR-A binding, while R-/IR-B and R^+^ cells were used for measurements on IR-B and IGF1R, respectively. A representative curve for each peptide to each receptor is shown. Each point represents the mean ± SEM of duplicates, and each experiment was repeated at least three times. **D-F:** Dose–response curves for ligand-induced autophosphorylation of IR-A and IGF1R. HEK293 cells overexpressing human IR-A **(D)** or IGF1R **(E and F)** were used. In **F**, ligands at indicated concentrations were co-incubated with 5 nM IGF-1. Each point represents the mean ± SEM of duplicates (n ≥ 2).

To characterize the new VILPs, we synthesized both single chain (sc, with the C-peptide) and double chain (dc, without the C-peptide) forms, resembling IGF-1 and insulin, respectively. Using a binding competition assay with ^125^I-Insulin and ^125^I-IGF-1, we demonstrated that all four VILPs competed with human insulin or IGF-1 for binding to IR-A/IR-B and IGF1R, respectively, with different affinities depending on the receptor type. The dissociation constants and relative binding affinities are summarized in Table 2 (IR-A and IR-B) and Table 3 (IGF1R), while representative binding competition curves are displayed in Figure 2A-C.

**Table 2:** **Receptor binding affinities of human insulin, human IGF-1 and VILPs to human IR-A and IR-B.** Binding affinity is reported by the equilibrium dissociation constant (K_d_). The K_d_ values were obtained from at least three independent measurements (indicated as n).

Ligand	IR-A	IR-B
	Kd [nM] ± S.D. (n)	Kd [nM] ± S.D. (n)
Human insulin	0.52 ± 0.03 (5)#	0.58 ± 0.07 (3)#
	0.32 ± 0.01 (3)$	0.38 ± 0.14 (3)$
Human IGF-1	105 ± 19 (4)#	176 ± 22 (3)#
scMFRV	211 ± 89 (3)#	565 ± 248 (3)
dcMFRV	68.1 ± 5.0 (3)$	537 ± 289 (3)
scLCDV-Sa	458 ± 205 (3)#	918 ± 98 (3)#
dcLCDV-Sa	487 ± 287 (5)#	994 ± 55 (4)#

The Kds of ligands were determined in two independent measumerements (# and $).

**Table 3:** **Receptor binding affinities of human insulin, human IGF-1 and VILPs to human IGF1R.** Binding affinity is reported by the equilibrium dissociation constant (K_d_). The K_d_ values were obtained from at least three independent measurements (indicated as n).

Ligand	IGF1R
	Kd [nM] ± S.D. (n)
Human IGF-1	0.24 ± 0.13 (3)#
	0.30 ± 0.06 (5)$
Human insulin	293 ± 101 (3)#
scMFRV	2.85 ± 0.09 (3)#
dcMFRV	16.3 ± 0.6 (3)$
scLCDV-Sa	60.8 ± 18.3 (3)#
dcLCDV-Sa	639 ± 453 (4)#

The Kds of ligands were determined in two independent measumerements (# and $).

The affinities of both forms of LCDV-Sa and scMFRV-VILP for both isoforms of IR were lower than IGF-1. However, the affinity of dcMFRV-VILP on IR-A was 1.5-fold stronger than IGF-1 (K_d_ 68 nM compared to 105 nM). Both forms of MFRV-VILP had slightly higher affinity to IR-A and IR-B than both forms of LCDV-Sa-VILP, with the difference being more profound on IR-A (Figure 2A,B, Table 2). While the affinities of sc and dcMFRV-VILP to IR-B were comparable (K_d_ 565 nM and 537 nM, respectively), dcMFRV-VILP had about 3-fold higher affinity to IR-A compared to the sc form (K_d_ 68 nM compared to 211 nM). The binding affinities of both forms of LCDV-Sa were comparable on both IR-A (K_d_ 458 nM and 487 mM for sc and dc, respectively) and IR-B (918 nM and 994 nM for sc and dc, respectively).

ScMFRV-VILP and dcMFRV-VILP bound to IGF1R with significantly higher affinity than human insulin (K_d_ 2.85 nM and 16.3 nM, compared to 293 nM for human insulin, Table 3). The same was observed for scLCDV-Sa-VILP, which had a ∼5-fold higher affinity for IGF1R than human insulin (K_d_ 60.8 nM). Both forms of MFRV-VILP showed higher affinity for IGF1R than both forms of LCDV-Sa-VILP. The scVILPs had higher affinity for IGF1R than the dc forms (∼5-fold higher for scMFRV-VILP and 10-fold higher for scLCDV-Sa-VILP). Additionally, scMFRV-VILP had an affinity only ∼10-fold weaker than IGF-1 (K_d_ 2.85 nM compared to 0.24 nM). We recently demonstrated that scLCDV-1-VILP is a natural antagonist of IGF1R and exhibits a comparable affinity to the MFRV-VILP. This establishes that these two VILPs possess significantly higher affinity for IGF1R compared to the other characterized VILPs [[11], [12], [13], [14]].

### 3.3 scMFRV-VILP reduces IGF-1 stimulated autophosphorylation of the IGF1R in high concentrations

We performed receptor phosphorylation assays to examine whether the binding potencies of VILPs are translated into signaling using HEK293 cells overexpressing either human IR-A or IGF1R. We stimulated the cells with increasing concentrations of the ligands for 15 min. The results of IR-A autophosphorylation were consistent with the binding affinities for LCDV-Sa VILPs and dcMFRV-VILP (Figure 2D, Table S4). ScMFRV-VILP stimulated IR-A autophosphorylation with a potency comparable to IGF-1 (EC_50_ ∼13.9 nM compared to ∼15.4 nM). Interestingly, this was about 10-fold higher than dcMFRV-VILP (EC_50_ ∼151 nM), despite a 3-fold weaker binding affinity for IR-A (K_d_ 211 nM, compared to 68 nM).

The results of IGF1R autophosphorylation were in line with the binding competition findings. ScMFRV-VILP showed potency similar to IGF-1 (EC_50_ ∼1.19 nM and 1.36 nM, respectively). The other ligands, ranked in order of potency, were dcMFRV-VILP (EC_50_ ∼14.5 nM), scLCDV-Sa-VILP (EC_50_ ∼19.3 nM), insulin, and dcLCDV-Sa-VILP (EC_50_ ∼282 nM) (Figure 2E, Table S4). While IGF-1 and scMFRV-VILP produced bell-shaped dose–response curves, the other peptides including insulin produced sigmoidal curves (Figure 2E). Interestingly, although scMFRV-VILP and IGF-1 had similar EC_50_ values, the maximum response of scMFRV-VILP was only ∼80 % of IGF-1. Furthermore, scMFRV-VILP inhibition of receptor autophosphorylation occurred at a 5-fold lower concentration than for IGF-1 (IC_50_ ∼313 nM compared to ∼1546 nM).

To investigate further, we examined whether scMFRV-VILP could inhibit IGF-1 stimulated IGF1R autophosphorylation in the IGF1R overexpressing HEK 293 cells. We treated the cells with increasing concentrations of scMFRV-VILP along with 5 nM IGF-1 and compared the results with those obtained with IGF-1, dcMFRV-VILP, and both forms of LCDV-Sa-VILP. While none of the other ligands showed any inhibitory effects, scMFRV-VILP and IGF-1 could inhibit IGF-1 stimulated IGF1R autophosphorylation at high concentrations (Figure 2F). Notably, scMFRV-VILP was 6-fold more potent than IGF-1, with an IC_50_ of ∼245 nM compared to ∼1484 nM for IGF-1 (Table S2).

### 3.4 ScMFRV-VILP specifically inhibits Erk phosphorylation but not Akt phosphorylation via IGF1R

**Figure 3:**
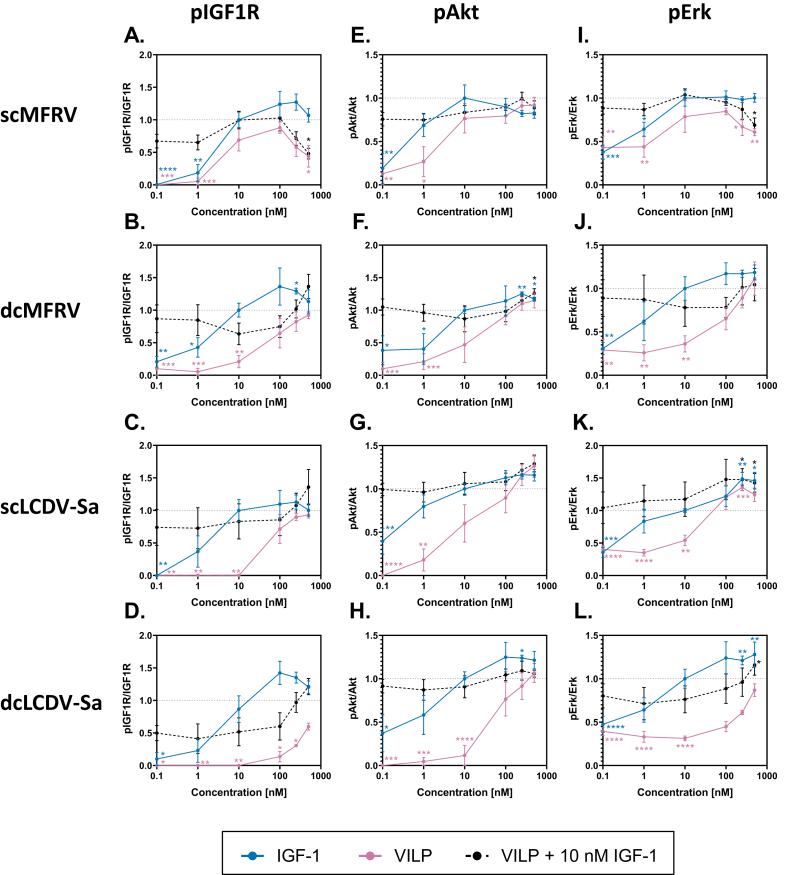
**Quantification of insulin/IGF signaling via IGF1R stimulated by MFRV and LCDV-Sa-VILPs.** R^+^ cells were treated with human IGF-1, VILP, or a combination of VILP and 10 nM IGF-1, and the phosphorylation levels of IGF-1R, Akt, and Erk1/2 were evaluated after 30 min. The data points represent the mean ± SEM of signals quantified from at least three independent experiments, expressed as a fold change relative to10 nM IGF-1 (grey dashed line). Each data point corresponds to the signal of the phosphorylated protein normalized to the total protein. Statistical significance was determined by unpaired two-tailed Student's t-test (∗P < 0.05, ∗∗<0.01, ∗∗∗P < 0.001, ∗∗∗∗P < 0.0001).

**Figure 4:**
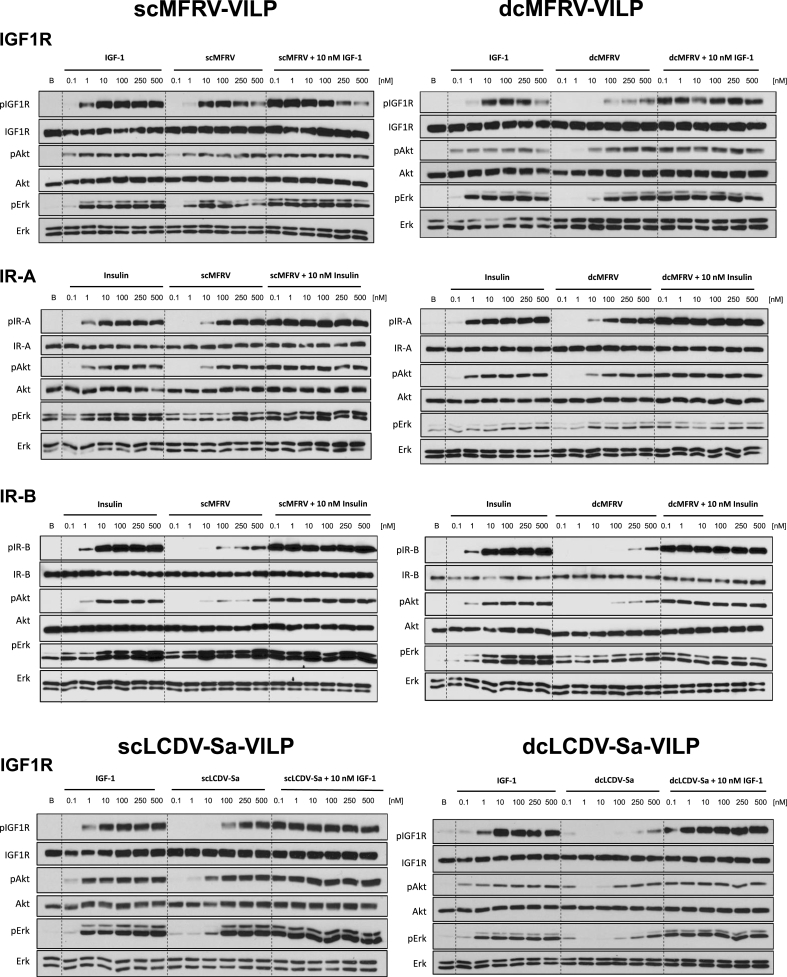
**Western blot analysis of MFRV and LCDV-Sa stimulated insulin/IGF signaling on human IR-A, IR-B, and IGF1R.** R-/IR-A, R-/IR-B or R^+^ cells were used for measurements on human IR-A, IR-B or IGF1R, respectively. Cells were stimulated with increasing concentrations of insulin (IR-A and IR-B) or IGF-1 (IGF1R), VILP and VILP in combination with 10 nM insulin/IGF-1 depending on the cell line. Phosphorylation of the receptor, Akt and Erk1/2, as well as the relative amounts of the total proteins, were observed in 30 min after stimulation. Representative western blots are shown, each experiment was repeated at least three times.

To determine the effects of scMFRV-VILP stimulated inhibition of IGF1R autophosphorylation in downstream signaling, we conducted insulin/IGF signaling experiments using IGF1R overexpressing mouse embryonic fibroblasts (R^+^ cells) [21]. The cells were stimulated either with human IGF-1, each form of LCDV-Sa and MFRV-VILPs, or a combination of VILPs and 10 nM IGF-1 in a dose–response manner (0.1 nM–500 nM). We measured the phosphorylation of IGF1R, Akt (PI3K/Akt pathway), and Erk1/2 (Ras/MAPK pathway). The quantifications of multiple experiments are shown in Figure 3 and representative western blots are shown in Figure 4. All VILPs stimulated IGF1R autophosphorylation with a potency lower than that of human IGF-1. ScMFRV-VILP showed the highest potency among the VILPs tested, followed by dcMFRV-VILP, scLCDV-Sa-VILP and dcLCDV-Sa-VILP. Consistent with the phosphorylation assay, scMFRV-VILP generated a bell-shaped dose response curve of IGF1R phosphorylation with a maximum at 100 nM. All other VILPs generated sigmoidal curves (Figure 3A–D, Figure 4). At 500 nM concentration, scMFRV VILP effectively inhibited IGF-1 stimulated autophosphorylation of IGF1R (Figures 3A and 4). We observed a similar trend for scMFRV-VILP at 250 nM concentration. In contrast, other VILPs tested did not exhibit any inhibitory effect on IGF1R autophosphorylation. Therefore, this was a specific effect of the scMFRV-VILP (Figure 4B-D).

All VILPs stimulated Akt and Erk phosphorylation in accordance with receptor autophosphorylation data (Figure 3E–H, 4). However, scMFRV-VILP did not inhibit IGF-1 stimulated Akt phosphorylation at high concentrations. In contrast, the phosphorylation of Erk stimulated by the VILP alone decreased when concentrations reached 250 nM and higher. Additionally, 500 nM scMFRV-VILP exhibited a significant inhibitory effect on the Erk phosphorylation stimulated by 10 nM IGF-1 (Figure 3I. 4). This indicates that scMFRV-VILP is a biased inhibitor, specifically inhibiting IGF1R and Erk phosphorylation (MAPK/Erk pathway) at high concentrations but not Akt phosphorylation (PI3K/Akt pathway). Neither form of LCDV-Sa VILP (Figure 3C, **D**, **G**, **H**, **K**, **L** and Figure 4) nor dcMFRV-VILP (Figure 3B, **F**, **J** and Figure 4) exhibited similar inhibitory effects. The difference in inhibitory functions of scMFRV and dcMFRV-VILPs on IGF1R indicates the crucial role of the C-domain in the inhibitory mechanism.

### 3.5 ScMFRV-VILP does not inhibit IR autophosphorylation

**Figure 5:**
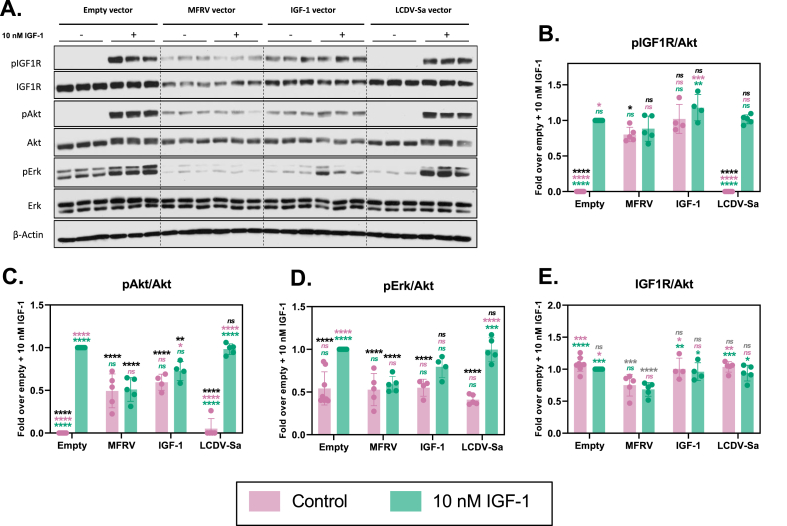
**Transfection of R**^**+**^**cells with VILPs or IGF-1 reveals unique effects of MFRV-VILP on IGF1R protein levels**. **A:** Representative western blot of IGF1R, Akt and Erk1/2 phosphorylation, as well as the relative amounts of total IGF1R in transfected R^+^ cells stimulated with 10 nM IGF-1 for the final 30 min of a 35 h transfection. **B:** Quantification of IGF1R phosphorylation, **C:** Quantification of Akt phosphorylation, **D:** Quantification of Erk1/2 phosphorylation, **E:** Quantification of total IGF1R. Each data point in the quantifications represents the mean ± SD of signals quantified from at least four independent experiments. Ordinary one-way ANOVA followed by Tukey's multiple comparison test was applied (∗P < 0.05; ∗∗P < 0.01, ∗∗∗P < 0.001, ∗∗∗∗P < 0.0001). The statistics are shown as each group is compared to the following: (i) control group (empty vector), represented in grey; (ii) empty vector + 10 nM IGF-1, represented in black; (iii) MFRV vector, represented in pink; and (iv) MFRV vector + 10 nM IGF-1, represented in green. (For interpretation of the references to color in this figure legend, the reader is referred to the Web version of this article.)

**Figure 6:**
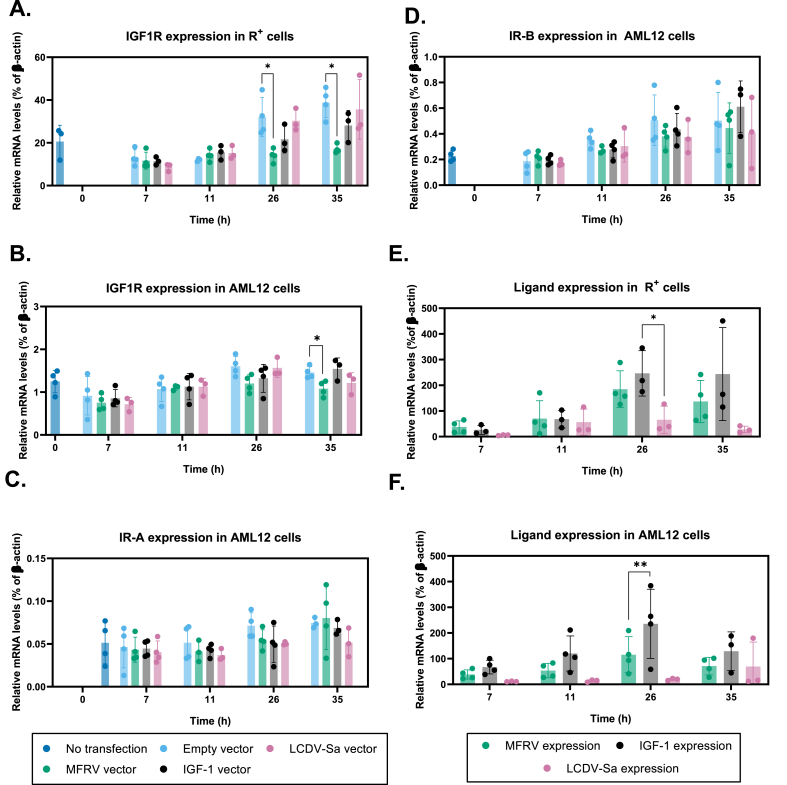
**MFRV-VILP transfection decreases IGF1R gene expression but does not affect IR.** Cells were transfected with empty vector or vectors carrying MFRV-VILP, human IGF-1, or LCDV-Sa-VILP. Gene expression was analyzed at different time points post-transfection (0, 11, 26, and 35 h) under serum-starved conditions. **A** and **E:** R^+^ cells, **B-D** and **F**: AML12 cells. Gene expression data are presented as a percentage of β-actin and represented as mean ± SEM n = 3–4). Ordinary one-way ANOVA followed by Tukey's multiple comparison test was applied to determine the statistical significance of the differences between groups (∗P < 0.05; ∗∗P < 0.01; ∗∗∗P < 0.001; ∗∗∗∗P < 0.0001).

To assess whether the scMFRV-VILP stimulated inhibition of IGF1R autophosphorylation and Erk phosphorylation was specific to IGF1R, and to define the effects of these VILPs on the IR, we conducted a series of signaling experiments using the IR-A or IR-B overexpressing mouse embryonic fibroblasts (R^−^/IR-A and R^−^/IR-B cells). We stimulated the cells with increasing concentrations of either human insulin, VILPs or mixtures of increasing concentrations of the VILP with 10 nM insulin. The quantifications of multiple experiments are shown in Figure 4 (IR-A) and Figure 5 (IR-B) and representative western blots are shown in Figure 4 and Figure 6.

All VILPs stimulated IR-A autophosphorylation and both forms of LCDV-Sa-VILP and dcMFRV-VILP had similar, slightly lower potencies in IR-A autophosphorylation compared to scMFRV VILP (Figure 4, S4A-D, S6). In terms of Akt phosphorylation, all VILPs showed dose-dependent stimulation (Figure 4, S4E-H, S6), with both forms of MFRV-VILP showing similar potency. DcLCDV-Sa-VILP was slightly less potent, and scLCDV-Sa-VILP was the weakest ligand among the VILPs. ScMFRV-VILP was the most potent VILP in Erk phosphorylation via IR-A, with the signal detected at 250 nM significantly exceeding that stimulated by 10 nM insulin (Fig. S4I). DcMFRV-VILP and both forms of LCDV-Sa VILP stimulated Erk phosphorylation with similar, slightly lower potencies. The VILPs did not inhibit Akt or Erk phosphorylation stimulated by 10 nM insulin (Figure 4, S4J-L, S6).

All VILPs stimulated IR-B autophosphorylation, but with lower potency than human insulin (Figure 4, S5A-D and S6) and they were less potent on IR-B compared to IR-A. ScMFRV-VILP was the most potent with ∼80 % maximum response compared to 10 nM insulin, and a signal detectable at 100 nM (Fig. S5A). DcMFRV-VILP had a comparable maximum response but with almost no detectable autophosphorylation at 100 nM (Figure 4, S5B). Both forms of LCDV-Sa-VILP reached lower maximum response (∼60-70 % of 10 nM insulin) but a signal was detectable at 100 nM (∼20-30 % response of 10 nM insulin). Akt phosphorylation was consistent with IR-B autophosphorylation. Both forms of MFRV-VILP were more potent in Akt phosphorylation than both forms of LCDV-Sa-VILP. VILPs were less potent or comparable to 10 nM insulin in Erk phosphorylation. None of the peptides inhibited phosphorylation of IR-B or downstream Akt or Erk at any concentration, except for scLCDV-Sa-VILP at 1 nM, which decreased Erk phosphorylation (Fig. S5K); however, further experiments are needed to confirm this effect.

Taken together, our findings demonstrate that while scMFRV-VILP is an agonist at IGF1R producing autophosphorylation in lower concentrations, it can also inhibit IGF-1 stimulated IGF1R phosphorylation and subsequent Erk activation at higher concentrations (>100 nM). Importantly, this inhibitory effect is specific to IGF1R and does not impact IR signaling.

### 3.6 MFRV-VILP transfection of cells decreases total IGF1R levels and inhibits Erk phosphorylation

We next examined the effects of prolonged exposure to MFRV-VILP on IGF1R signaling by transfecting the IGF1R overexpressing R^+^ cells either with MFRV-VILP, human IGF-1, LCDV-Sa-VILP, or an empty vector. After 34.5 h, the cells were stimulated with 10 nM IGF-1 for the final 30 min of the transfection (no stimulation was performed in the control group). MFRV-VILP and IGF-1 transfections induced comparable autophosphorylation of IGF1R, while the transfections with the empty vector and LCDV-Sa-VILP had no effect (Figure 5A,B). MFRV-VILP transfection led to significantly lower receptor autophosphorylation than IGF-1 transfection in response to IGF-1 stimulation indicating MFRV-VILP's inhibitory effects (Figure 5B). Additionally, both MFRV-VILP and IGF-1 transfections led to significantly reduced IGF-1 stimulated Akt phosphorylation compared to empty vector transfection (Figure 5C). Only MFRV-VILP transfection reduced IGF-1 stimulated Erk phosphorylation (Figure 5D), consistent with previous experiments. The most notable observation was the significant decrease in total IGF1R levels after MFRV transfection (Figure 5A,E), which was specific to MFRV-VILP and not observed with IGF-1 or LCDV-Sa transfection.

### 3.7 MFRV-VILP downregulates IGF1R gene expression

To determine whether the downregulation of IGF1R protein levels was caused by decreased gene expression, we performed another transfection experiment using IGF1R overexpressing R^+^ cells and collected samples at pre (t0) and post-transfection (7 h, 11 h, 26 h and 35 h). IGF1R gene expression was significantly decreased in MFRV-VILP transfected R^+^ cells compared to empty vector transfection at both 26 h and 35 h post-transfection. Neither IGF-1 nor LCDV-Sa-VILP transfection had a similar effect (Figure 6A), indicating the specificity of the MFRV-VILP function. To control for potential effects of overexpressing IGF1R, we repeated the transfection experiments in AML12 hepatocytes and observed a trend of decrease in IGF1R expression in MFRV-VILP transfected cells at 26 h post-transfection and a significant decrease at 35 h. IGF-1 and LCDV-Sa-VILP transfection did not affect IGF1R gene expression (Figure 6B). We also assessed IR-A and IR-B expression in AML12 hepatocytes and did not observe any significant changes in expression of these receptors after transfection with any of the ligands compared to transfection with empty vector (Figure 6C,D). We also tested ligand expression in both R^+^ (Figure 6E) and AML12 cells (Figure 6F) and showed that the expression of all the ligands reached maximum in 26 h post-transfection.

To determine whether MFRV-VILP is secreted into the medium after transfection, we first examined the presence of a signal peptide in MFRV VILP. SignalP analysis predicted the existence of a signal peptide with a high score (0.9907, Fig. S7A). To validate this finding experimentally, we conducted a supernatant transfer experiment. For this purpose, AML12 cells were initially transfected with either MFRV-VILP or an empty vector, as detailed earlier, and subsequently, the supernatants were transferred to serum-starved AML12 cells. The supernatant obtained from cells transfected with MFRV-VILP induced receptor, Akt, and Erk phosphorylation, while no signaling stimulation was observed in cells stimulated with the supernatant obtained from cells transfected with the empty vector (Fig. S7B). We subsequently investigated the impact of chemically synthesized MFRV-VILP on IGF1R gene expression. To assess this, we stimulated R+ cells overexpressing IGF1R with 100 nM of either MFRV-VILP or IGF-1 at 3-h intervals over a 24-hour period. Interestingly, scMFRV-VILP significantly reduced IGF-1R gene expression (∼40 % decrease). In contrast, neither dcMFRV-VILP nor IGF-1 demonstrated a similar effect (Fig. S7C). Overall, our results indicate that the effects of MFRV-VILP on IGF1R gene expression is unique and can be observed not only in IGF1R overexpressing R^+^ cells, but also in a natural cell line, AML12 hepatocytes.

### 3.8 MFRV-VILP inhibits IGF-1 stimulated cell proliferation in vitro and lowers blood glucose in vivo

**Figure 7:**
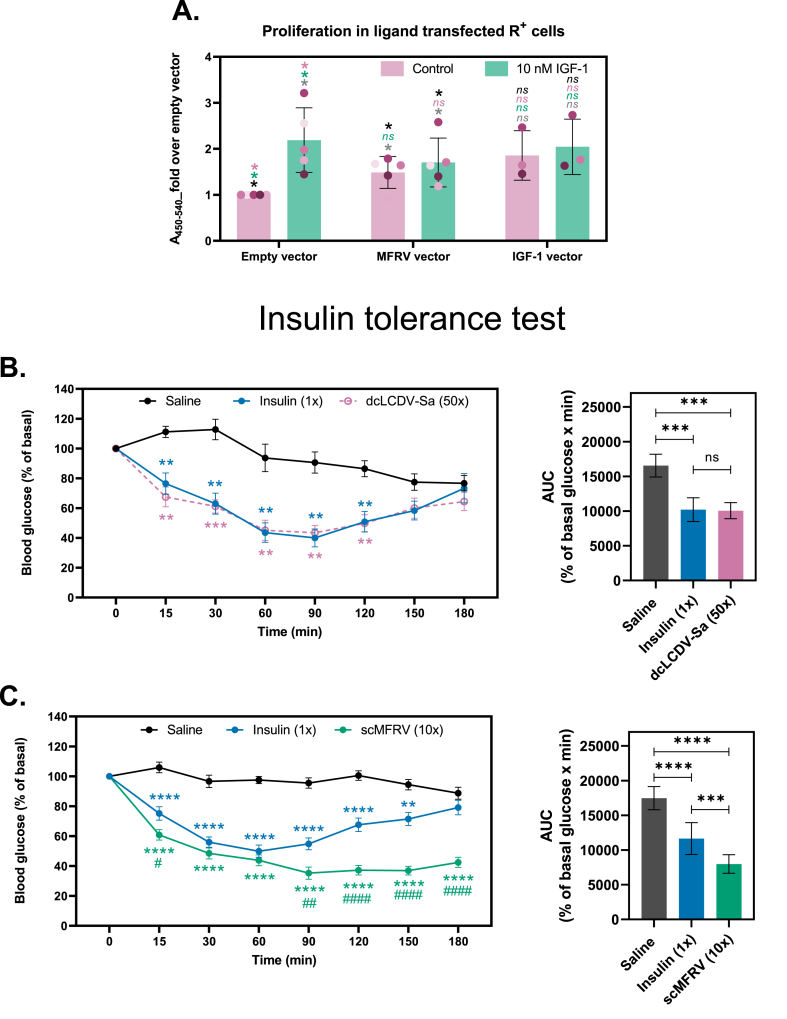
**MFRV-VILP decreases IGF-1 stimulated proliferation in vitro while stimulates long-lasting blood glucose lowering effects in vivo. A:** Proliferation assay of R^+^ cells transfected with MFRV-VILP and IGF-1. Cells were transfected with empty vector or vectors carrying MFRV-VILP or human IGF-1, serum-starved for 24 h post-transfection and incubated or not (control) with 10 nM IGF-1 for an additional 24-hour period starting 24 h post-starvation. Incorporation of BrdU was assessed to measure cell proliferation. Results are expressed as fold over empty vector. Data points from the same experiment are labeled in identical color. A paired two-tailed Student's t-test was performed (∗P < 0.05). The statistics are shown as each group is compared to the following: (i) control group (empty vector), represented in grey; (ii) empty vector + 10 nM IGF-1, represented in black; (iii) MFRV vector, represented in pink; and (iv) MFRV vector + 10 nM IGF-1, represented in green. **B** and **C**: Insulin tolerance test. C57BL/6 J mice were injected i.p. with human insulin, dcLCDV-Sa-VILP, scMFRV-VILP, or saline. The insulin concentration was 6 nmol/kg in both panels, whereas the concentration of dcLCDV-Sa-VILP was 0.3 μmol/kg **(B)** and the concentration of scMFRV-VILP was 60 nmol/kg **(C)**. Blood glucose was measured within the range of 0–180 min. Data are mean ± S.E.M. Mixed-effects analysis followed by Dunnett's multiple comparisons test was applied, n = 4–11 per condition (∗P < 0.05; ∗∗P < 0.01, ∗∗P < 0.001). Comparisons were made to saline (**∗**), or human insulin **(#)**. The area under the curve for each condition is shown on the right side of each panel. Ordinary one-way ANOVA followed by Tukey's multiple comparison test was applied for statistical analysis (∗P < 0.05; ∗∗P < 0.01, ∗∗∗P < 0.001, ∗∗∗∗P < 0.0001). (For interpretation of the references to color in this figure legend, the reader is referred to the Web version of this article.)

We evaluated the effect of MFRV-VILP on cell growth by transfecting IGF1R overexpressing R^+^ cells with different vectors (VILPs or IGF-1), followed by serum-starvation and stimulation with either 10 nM IGF-1 or serum-free medium. We assessed cell proliferation using the BrdU probe during the final 6 h of stimulation. 10 nM IGF-1 stimulation resulted in a 2.2-fold increase in cell proliferation in cells transfected with empty vector (Figure 7A). On the other hand, MFRV-VILP alone increased the cell proliferation by 1.5-fold compared to the empty vector, and 10 nM IGF-1 stimulation did not further increase proliferation in MFRV-VILP transfected cells. These observations suggest that while MFRV-VILP alone enhances cell proliferation, it inhibits IGF-1 stimulated proliferation, which is consistent with our prior findings.

To further investigate the functional properties of VILPs in vivo, we conducted an insulin tolerance test (ITT). We administered different doses of dcLCDV-Sa-VILP, dcMFRV-VILP, and scMFRV-VILP to C57BL/6 J mice (n = 4–11), along with saline or 6 nmol/kg insulin as controls (Figure 7B, Fig. S8). Due to the higher affinity of MFRV-VILPs for IR-A and IGF1R compared to LCDV-Sa-VILPs, we used a 0.3 μmol/kg dose (50x higher concentration than human insulin) for dcLCDV-Sa-VILP and a 60 nmol/kg dose (10x compared to insulin) for both forms of MFRV-VILPs. As previously reported [11,12], insulin caused a maximum decrease of ∼60 % in blood glucose levels, which gradually returned to normal (Figure 7B,C, and S7). The glucose-lowering effect of 50x dcLCDV-Sa-VILP was comparable to insulin (Figure 7B). More importantly, 10x scMFRV-VILP demonstrated a potent and sustained decrease in blood glucose levels, with a maximum reduction of 65 % that persisted for 3 h post-injection (Fig. 7C). Based on this result, we tested the same dose of scMFRV-VILP as insulin (6 nmol/kg, 1x), which demonstrated a significant but weaker blood glucose-lowering effect (maximum of 20 %) compared to insulin (Fig. S8B). However, scMFRV-VILP (1x) produced a similar long-acting effect, as blood glucose levels remained stable up to 180 min post-injection. In contrast, dcMFRV-VILP (10x) produced a comparable effect to scMFRV-VILP (1x) and glucose levels gradually normalized. We previously completed similar ITT experiments using IGF-1 and we did not observe similar long-lasting effects [12].

### 3.9 CryoEM modeling of the IGF1R ectodomain in complex with scMFRV-VILP reveals that the VILP binds to the receptor in a similar manner to IGF-1

**Figure 8:**
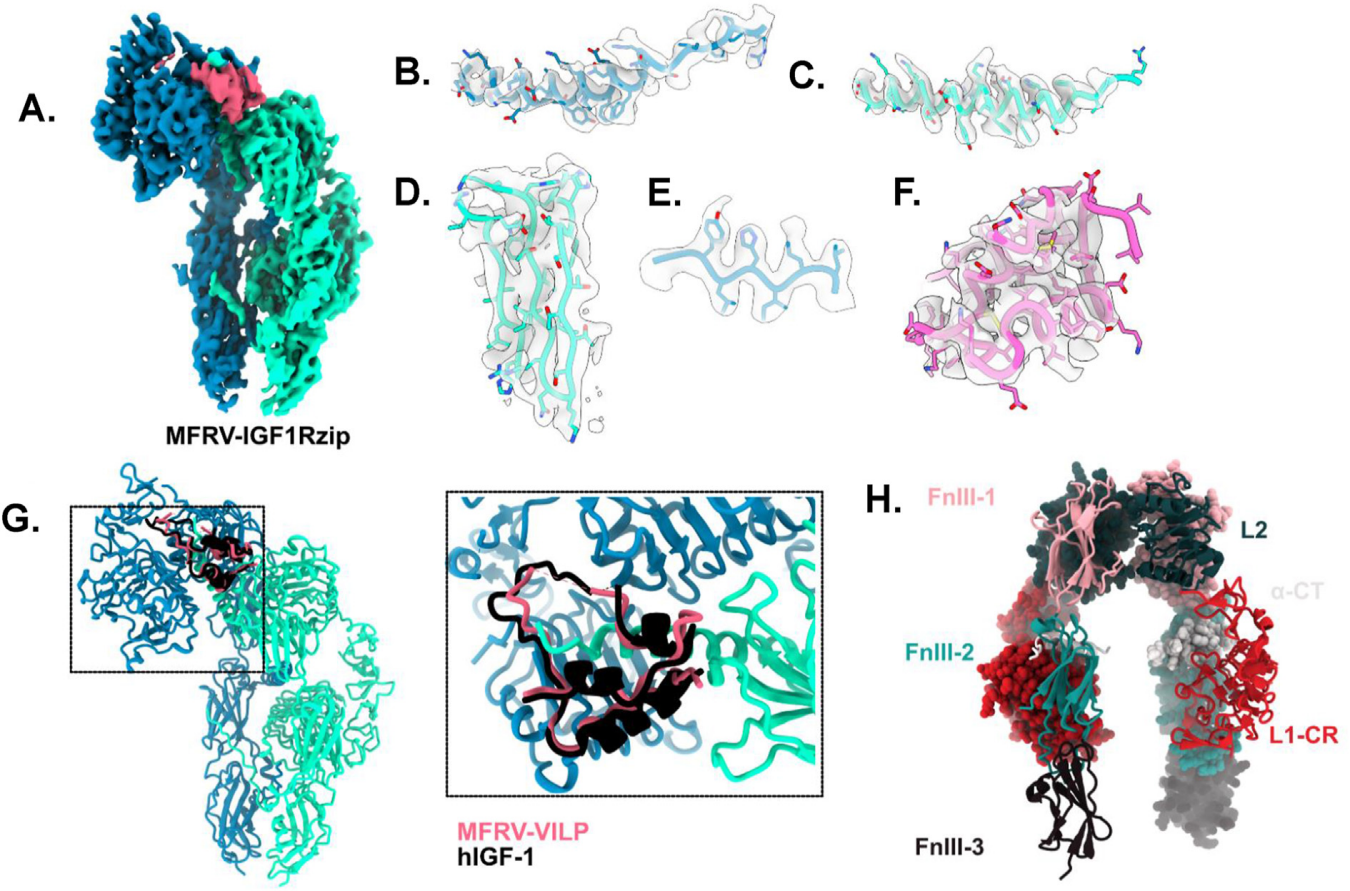
**scMFRV- VILP-IGF1Rzip cryoEM reconstruction. a.** cryoEM density after 3D-flexible refinement of MFRV-IGF1Rzip, colored by chain. **b-f.** Density associated with refined model: **b.** ɑCT. **c.** ɑCTʹ. d. FnIII-1ʹ. **e.** L1. **f.** MFRV-VILP. g. overlay of hIGF-1 (black) from hIGF1-mIGF1R (pdb: 6PYH) with MFRV-IGF1Rzip with zoomed and reoriented inlay. h. apo-IGF1R with domains labelled (from PDB: 5U8R).

To determine whether the altered effect of scMFRV-VILP on human IGF1R signaling was reflected in a structural change of the receptor complex, we performed cryo-electron microscopy of IGF1Rzip, a human IGF1R ectodomain construct, in the presence of varying stoichiometric ratios of scMFRV-VILP and human IGF-1. Reconstructions were readily interpretable from the initial model stage in each case. With a 4-fold excess of scMFRV-VILP (calculated per receptor αβ monomer), two major conformations were observed: an open-leg and a closed-leg as previously described for human IGF-2 bound structures [28]. This result was also observed with a 1:1 stoichiometric ratio of scMFRV-VILP to receptor and with a 1:1:1 stoichiometric ratio of scMFRV-VILP to IGF1 to IGF1Rzip (data not included). While the open-leg conformation was readily interpretable, a high-resolution reconstruction was not achieved, attributed to continual heterogeneity. For the closed-leg conformation, a 3.06 Å structure consisting of the intact ectodomain and a single bound scMFRV-VILP molecule was determined. The resultant model and associated cryoEM potential density, hereafter referred to as IGF1Rzip-scMFRV-VILP, are shown in Figure 8, Fig. S9 and S10.

The structure of IGF1Rzip-scMFRV-VILP was observed to be analogous to previous structures of human IGF-1 and IGF-2 bound to IGF1R. As previously, the overall receptor conformation is seen to be an asymmetric Γ shape [37]. One molecule of scMFRV-VILP is observed engaging the receptor via the primary binding site L1-CR + αCT' module, relocated to the top of the Γ from its position abutting FnIII-2′ in the apo conformation. This relocation is accommodated by hinging around the L2 domain relative to the FnIII domains, resulting in the membrane-proximal FnIII-3 domains coming together, forming the bottom of the Γ together with the remaining L1′-CR' + αCT module. Only one ligand is observed binding to the receptor, as for IGF-1. The points of membrane entry are approximately 20 Å apart, like previous “closed” structures of the IGF1Rzip construct (c.f. 19 Å for human IGF-2, 39 Å for human IGF1-mIGF1R, 67 Å for human apoIGF1R and 90 Å for scLCDV1-VILP-IGF1Rzip) [28,37,38].

As for all liganded IGF1R structures, the scMFRV-VILP C-loop is threaded through by the αCT' helix. This helix is more extensive compared to the apo conformation and observed to run perpendicular to the beta-strands of the L1 domain as for all previous ligand-bound IGF1R structures [14,28,37]. Its pitch and register are identical to the human IGF1-holo-mIGF1R structure. Despite 34 % sequence similarity, the binding site of scMFRV-VILP is observed to be remarkably like that of human IGF-1 and -2 in the A-domain and B-domain. The C-domain is largely disordered, though the density corresponding to Tyr29 is still present. The B-domain C-terminus follows a slightly different path along the L1, oriented relatively more towards the center of the L1 surface, though the density is not resolved clearly. There were no changes observed significant enough to directly explain a difference in downstream signaling between scMFRV-VILP and IGF-1 at the receptor level.

## 4 Discussion

In the last five years, we have reported the discovery [11] and characterization [[11], [12], [13],^39^] of five novel virally-encoded VILPs. These VILPS are ∼30–50 % identical to human insulin and IGF-1 and some of them demonstrate atypical effects on the insulin/IGF system, thereby providing new insights into these critical signaling systems. For example, dcGIV-VILP induced two-fold higher glucose uptake in white adipose tissue (WAT) than human insulin in vivo [12]. By contrast, we showed that scLCDV-1-VILP, but not dcLCDV-1-VILP, is a natural antagonist of the IGF1R [13,14]. In this study, we performed a comprehensive characterization of two previously uncharacterized VILPs, MFRV and LCDV-Sa-VILPs. We first showed that sc and dc forms of the MFRV and LCDV-Sa-VILPs can bind to and activate both isoforms of human IR and IGF1R. They also stimulate downstream insulin/IGF signaling with different potencies. Therefore, they are new members of the insulin/IGF family of ligands.

It is noteworthy that scMFRV-VILP acts as a biased inhibitor, inhibiting Erk phosphorylation but leaving Akt phosphorylation unaffected. This makes it the first VILP to exhibit biased inhibition. We previously showed that scLCDV-1-VILP inhibits both pathways. The distinctive VILP traits potentially result from co-evolution of host and the pathogen to aid viral pathogenesis in the host [15,16], unlike the evolution of the host insulin/IGFs [40]. The PI3K/Akt signaling pathway of the host is utilized for protein synthesis and therefore essential for viral replication [41]. Moreover, certain viruses also employ PI3K/Akt signaling as a strategy to slow down apoptosis [42]. On the other hand, MAPK/Erk pathway is essential for T-cell activity and blocking MAPK/Erk severely impairs T-cell activation [43]. Therefore, scMFRV-VILP may have specifically evolved to target IGF1R and inhibit the MAPK/Erk pathway, thereby suppressing T-cell related immune response. However, it has also likely evolved to preserve the integrity of PI3K/Akt signaling ensuring adequate protein synthesis to promote viral replication.

In addition to this unique finding, the prolonged blood glucose lowering effect of the scMFRV-VILP is also an intriguing observation. It is well established that insulin exerts its blood glucose lowering effect by activating the PI3K/Akt pathway [44], which aligns with our findings that scMFRV-VILP stimulates, rather than inhibits, this pathway. The prolonged MFRV-VILP action was specific to the scMFRV-VILP and was not observed for the dcMFRV-VILP, indicating that the presence of C-domain is essential for this effect. We and others have previously shown that IGF-1 does not have a similar prolonged blood lowering effect [12,45]. Because MFRV-VILP and IGF-1 share only two residues in the C-domain (analogous to Gly30 and Tyr31 of IGF-1), the C-domain might be responsible for this difference. Further studies are necessary to elucidate the underlying mechanism(s) responsible for the extended half-life of scMFRV-VILP. This may be attributed to a decreased potency of insulin-degrading enzyme on the VILP, a unique interaction between scMFRV-VILP and some IGF-binding proteins, or altered kinetics of release from the injection site. Understanding these mechanisms could offer valuable insights for design of novel, long-lasting insulin analogs, ultimately providing enhanced convenience for diabetes patients.

Another interesting result of this study was the observation that MFRV-VILP transfection downregulates IGF1R on both gene expression and protein levels. We first showed this effect in the IGF1R overexpressing R^+^ cells and then confirmed these results using AML12 hepatocytes, indicating that the effect is conserved in a natural cell line. Given that the IGF1R gene in the engineered R+ cell line is governed by a constitutively active SV40 early promoter [22,46], the observed decrease in IGF1R mRNA levels across both cell lines may not be regulated at the transcriptional level but rather at the post-transcriptional stage. The decrease in the gene expression was specific to IGF1R, MFRV-VILP transfection did not affect IR gene expression in AML12 hepatocytes. In accordance with previous studies that have examined the regulatory influence of IGF-1 on IGF-1R in the brain [47] and C2C12 myoblasts [48], our investigation has generated significant new findings. Specifically, our research reveals that MFRV-VILP exhibits a remarkable ability to modulate IGF1R gene expression not only in AML12 hepatocytes, but also in cells with overexpressed IGF-1 receptors. Conversely, IGF-1 itself does not induce similar regulatory effects in these specific cellular contexts. Similarly, LCDV-Sa-VILP fails to elicit these effects, albeit it's worth noting that in this instance, the VILP was expressed at lower levels in both cell lines compared to MFRV-VILP and IGF-1 (Figure 6D,E). Our results from the prolonged exposure experiment using chemically synthesized MFRV-VILPs also demonstrate that the effect is mediated by the VILP-receptor interaction. The specificity of this effect to the sc version of MFRV-VILP (Fig. S7B) reveals the involvement of the C-peptide in this function. Notably, our findings indicate that MFRV-VILP is processed as a single-chain (IGF-1-like) peptide in our transfection experiments and is secreted by these cells.

These results carry notable implications, as IGF1R expression is frequently upregulated in a range of tumor types, including breast, prostate, colorectal, and lung tumors [[49], [50], [51]]. Gaining a comprehensive understanding of the underlying mechanisms governing receptor abundance manipulation can offer valuable insights for the development of precise tools to target and regulate IGF1R levels specifically in tumor cells. Previous studies have identified important factors including breast cancer gene-1 (BRCA1), p53, the Wilm's tumor protein-1 (WT1) and the von Hippel-Lindau gene (VHL) that play a key role in IGF1R gene expression [52]. In addition to these suppressors, certain stimulatory nuclear proteins also regulate IGF1R gene transcription [52]. A recent study focusing on various cancer cell lines indicated that phosphorylated IGF1R is transported to the nucleus where it acts as a transcriptional factor [53]. Our future studies will determine the underlying mechanism of gene expression manipulation.

Interestingly, scMFRV-VILP binds to IGF1R with a relatively high affinity (about 10x lower than IGF-1), while dcMFRV-VILP has about 6x lower affinity than scMFRV-VILP. This indicates the importance of the C-domain in receptor binding. The presence of the MFRV C-domain was also shown to be indispensable for the unique properties of the VILP described above. Because the MFRV-VILP C-domain shares only one residue (Tyr30, analogous to Tyr31 in IGF-1) implicated in IGF1R binding with IGF-1 [37,54] (Fig. 1A), we initially hypothesized that the receptor conformation induced by binding of scMFRV-VILP to IGF1R might be significantly different compared to IGF-1. However, the cryo-EM analysis did not reveal any significant differences in IGF1R binding between the two peptides. The only observed difference was the density corresponding to the C-domain, which was less ordered in the case of scMFRV-VILP compared to IGF-1 [28] and other known structures [14,28]. This implies that the MFRV-VILP C-domain doesn't engage with the CR pocket of IGF1R as deeply as previous ligands. It is plausible that this shallow engagement permits the binding of a second scMFRV-VILP or IGF-1 ligand at high concentrations, producing the observed bell-shaped dose–response curve, however we were not able to observe this directly. The cryoEM results confirmed that the biased inhibition observed for MFRV-VILP occurs via a different mechanism to that previously identified for scLCDV1-VILP [13,14].

Our results demonstrated that scMFRV-VILP exhibits a biased inhibitory function in Erk phosphorylation, without affecting Akt phosphorylation. Interestingly, previous studies reported the MAPK/Erk pathway's role in regulation of IR endocytosis. It was shown that Erk mediated serine phosphorylation of insulin receptor substrate (IRS) proteins enhances the interaction of IRS with the clathrin adaptor AP2M1, which in turn facilitates clathrin mediated IR endocytosis [55,56]. Therefore, inhibition of Erk could reduce IR endocytosis, prolonging metabolic signaling at the plasma membrane [55,56]. Even though IRS-1 (but not IRS-2) was shown to negatively regulate IGF1R endocytosis [57], similar mechanisms as for IR cannot be ruled out. This might potentially explain scMFRV-VILP's impact on Erk phosphorylation, but not Akt phosphorylation and the prolonged glucose lowering effect in mice. Further, Zinkle and Mohammadi proposed a threshold model for RTK signaling specificity [58], suggesting that distinct downstream signals are emerging from varying RTK dimer strength or stability. According to the model, metabolic outcomes would require a less stable dimer compared to what is needed for proliferation or differentiation. Even though IR and IGF1R exist as dimers in the ligand-free state and do not dimerize upon ligand binding like other RTKs, their transmembrane domains remain separated in the absence of ligand. Ligand binding, however, enables them to come together, leading to a conformational change that facilitates autophosphorylation of their cytoplasmic tyrosine kinase domains [38,59,60]. Hence, the threshold model may remain plausible. It is conceivable that scMFRV could induce a less stable state in the approximation of IGF1R tyrosine kinase compared to IGF-1 and potentially result in “more” metabolic rather than growth-promoting effects. Nonetheless, all these hypotheses require further investigation.

As we delve into the selective inhibitory impact of scMFRV-VILP on signaling pathways and the dissimilar antagonistic responses between LCDV-1 and LCDV-Sa, an intriguing question surfaces about the underlying determinants driving varied functions of these LCDV VILPs. We previously characterized LCDV-1 VILP and established that scLCDV-1 VILP is a natural competitive antagonist of IGF1R [13,14]. On the other hand, we showed that LCDV-Sa is a weak agonist of IR and IGF1R and did not observe any distinct effects for the LCDV-Sa VILP. There are currently five Lymphocystis disease virus genomes available with three of them (LCDV-1, LCDV-4 and LCDV-Sa) possessing VILPs, indicating evolutionary divergence [61]. LCDV-1 was isolated from a fish obtained in the North-Sea while LCDV-Sa originated from Mediterranean fish farms suggesting geographical distance that is potentially affecting the evolution of these two isolates. Although these two VILPs share 39 % amino acid sequence identity overall, with 50 % in the A-chain and 43 % in the B-chain, there are no shared amino acids in the C-domain (Table 1). Our previous studies demonstrated the essential role of the C-domain in LCDV-1 for IGF1R inhibition, possibly accounting for the lack of antagonism observed in LCDV-Sa.

## 5 Conclusions

In this work, we have described and characterized four new members of the insulin/IGF family of hormones. Both MFRV and LCDV-Sa-VILPs (sc and dc) have demonstrated their ability to bind to IR and IGF1R, stimulate downstream signaling and reduce blood glucose levels in mice. In many aspects, scMFRV-VILP exhibits properties of an IGF-1 like ligand, which is demonstrated by the relatively high affinity of the VILP to IGF1R, the bell-shaped curve of IGF1R phosphorylation and the similar manner of binding of scMFRV-VILP to the receptor. However, we have also identified distinct and unique functions of scMFRV-VILP when compared to IGF-1. These include inhibition of Erk (but not Akt) phosphorylation in concentrations ≥250 nM, downregulation of IGF1R expression and prolonded glucose lowering effect in mice. These effects were specific to scMFRV-VILP and were not observed for dcMFRV-VILP, highlighting the importance of the C-domain. The result of cryoEM analysisdemonstrated that scMFRV-VILP binds to IGF1R in a manner similar to IGF-1, implied that its inhibitory and biased signaling effects manifest at the post-receptor signaling level. In summary, our study unveils MFRV and LCDV-Sa-VILPs as novel members of the insulin/IGF superfamily with scMFRV-VILP displaying distinct properties that set it apart from the native ligands. These results provide important insights into IGF1R function, inhibition and signaling, that can potentially guide the design of IGF1R inhibitors and the advancement of long-lasting insulin formulations.

## CRediT authorship contribution statement

**Martina Chrudinová:** Conceptualization, Data curation, Formal analysis, Investigation, Methodology, Writing – original draft, Writing – review & editing. **Nicholas S. Kirk:** Data curation, Formal analysis, Investigation, Methodology, Writing – original draft, Resources, Software. **Aurelien Chuard:** Data curation, Investigation. **Hari Venugopal:** Data curation, Formal analysis. **Fa Zhang:** Data curation, Formal analysis, Investigation, Methodology. **Marta Lubos:** Data curation, Formal analysis, Methodology. **Vasily Gelfanov:** Data curation, Formal analysis, Investigation. **Terezie Páníková:** Data curation, Formal analysis, Investigation. **Lenka Žáková:** Data curation, Formal analysis, Investigation, Methodology. **Julianne Cutone:** Data curation. **Matthew Mojares:** Data curation. **Richard DiMarchi:** Conceptualization, Funding acquisition, Supervision, Writing – original draft, Resources. **Jiří Jiráček:** Data curation, Formal analysis, Investigation, Methodology, Resources, Supervision, Writing – original draft. **Emrah Altindis:** Conceptualization, Data curation, Formal analysis, Funding acquisition, Investigation, Methodology, Project administration, Resources, Software, Supervision, Validation, Writing – original draft, Writing – review & editing.

## Declaration of competing interest

VG and RM are or were employees at Novo Nordisk. The other authors have no conflicts of interest to disclose related to this project.

## Data Availability

CryoEM map and atomic model of scMFRV-VILP bound to IGF1R.zip generated in this study have been deposited in the EMDB and PDB databases, respectively, under the accession codes EMD-41138and 8TAN.
