# Supplementary material for: A viral insulin-like peptide inhibits IGF-1 receptor phosphorylation and regulates IGF1R gene expression
Source: Mol Metab. 2024 Jan 3;80:101863. doi: 10.1016/j.molmet.2023.101863 (PMC10831276; doi:10.1016/j.molmet.2023.101863)
Supplement: Multimedia component 1 [file mmc1.pdf]

A.

Chemical Formula: $C_{295}H_{480}N_{78}O_{91}S_7$
Molecular Weight: 6800.0

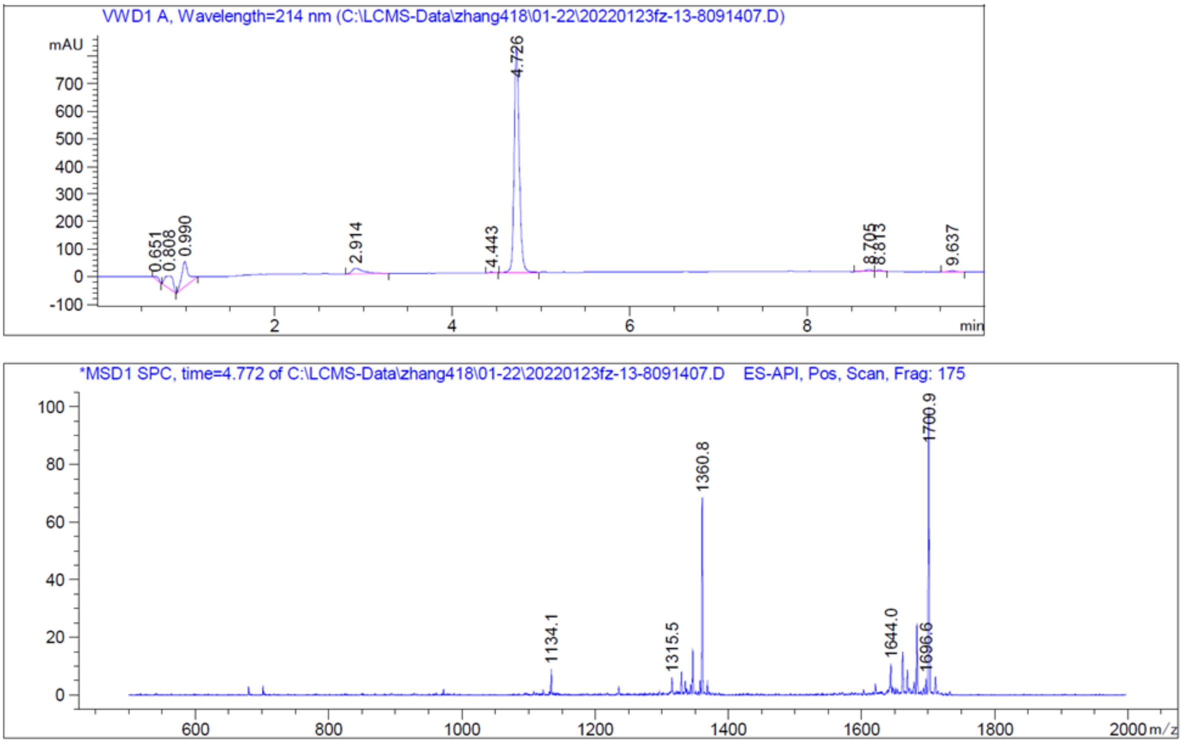

B.

Chemical Formula: $C_{296}H_{476}N_{80}O_{94}S_7$
Molecular Weight: 6883.95

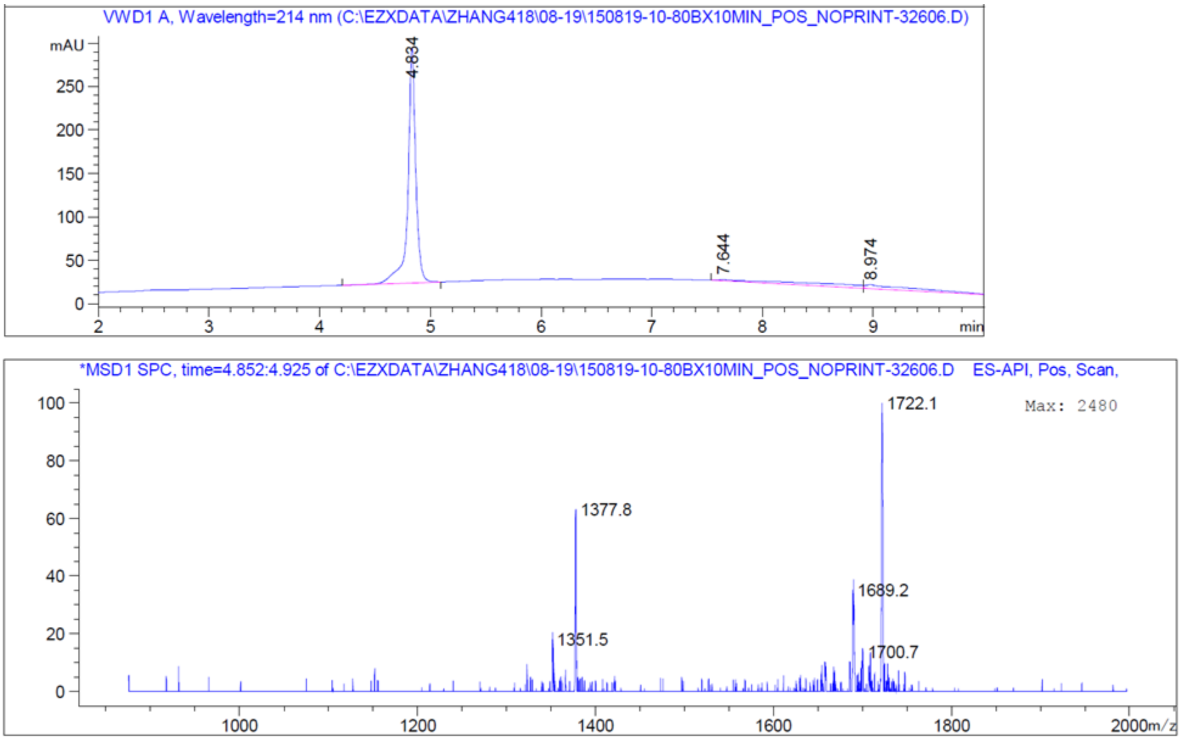

Figure S1: LC-MS spectra of synthesized scMFRV-VILP (A) and scLCDV-Sa-VILP (B).

Chemical Formula: $C_{249}H_{397}N_{63}O_{78}S_7$
Exact mass: 5741.7080
Molecular weight: 5745.6980

A.

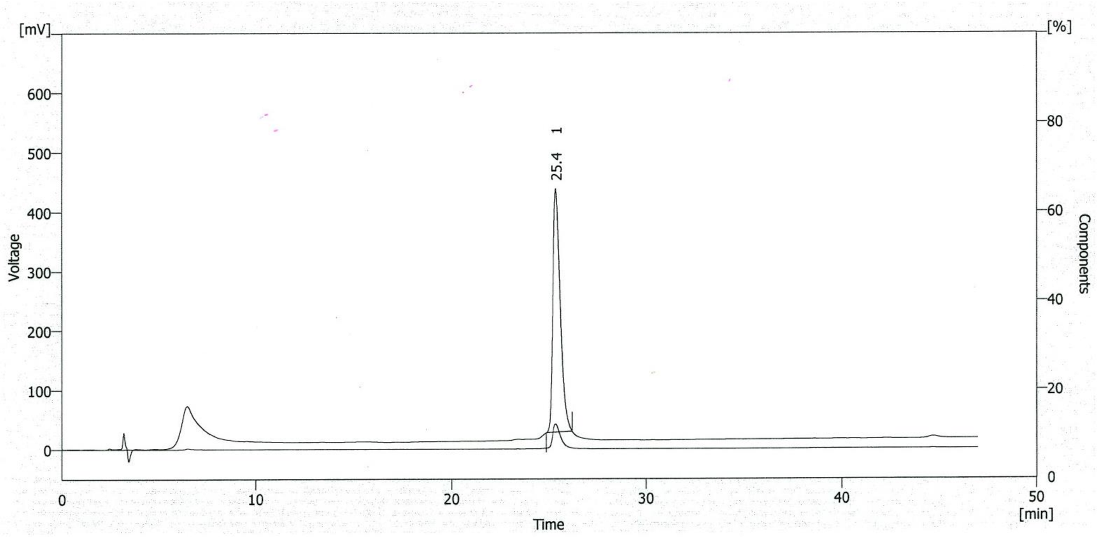

B.

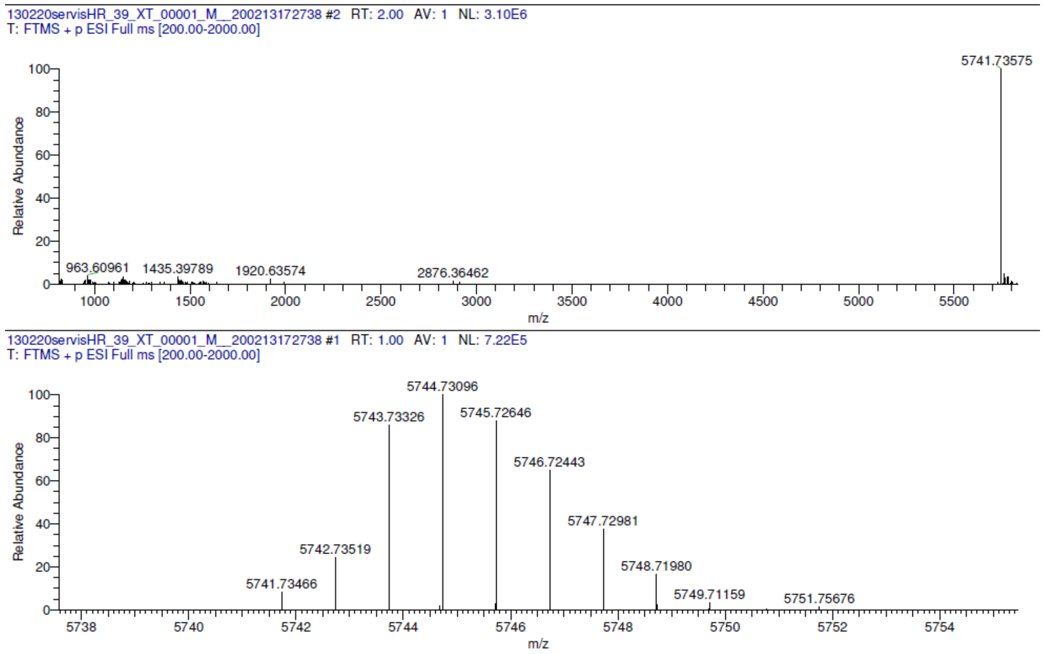

Figure S2: HPLC analysis (A) and HR-MS spectrum (B) of synthesized dcLCDV-Sa-VILP

Chemical Formula: $C_{247}H_{402}N_{62}O_{78}S_7$
Exact mass: 5708.7441
Molecular weight: 5712.7090

A.

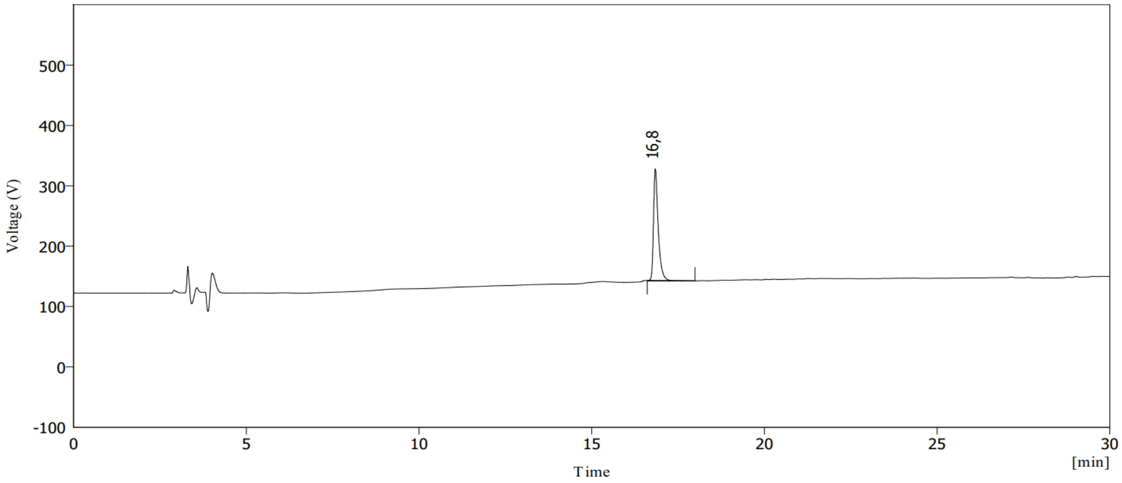

B.

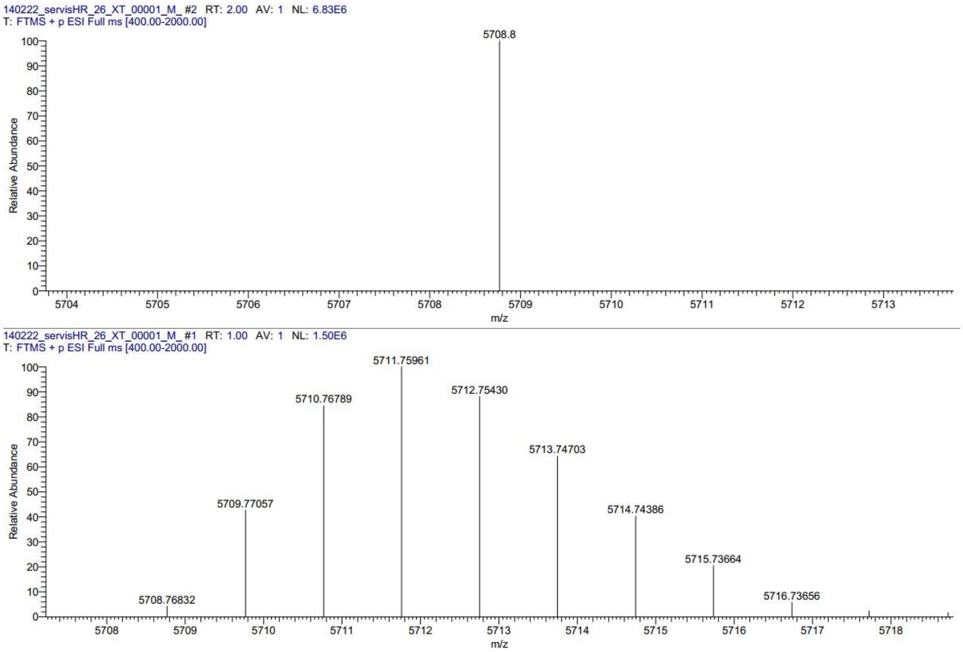

Figure S3: HPLC analysis (A) and HR-MS spectrum (B) of synthesized dcMFRV-VILP

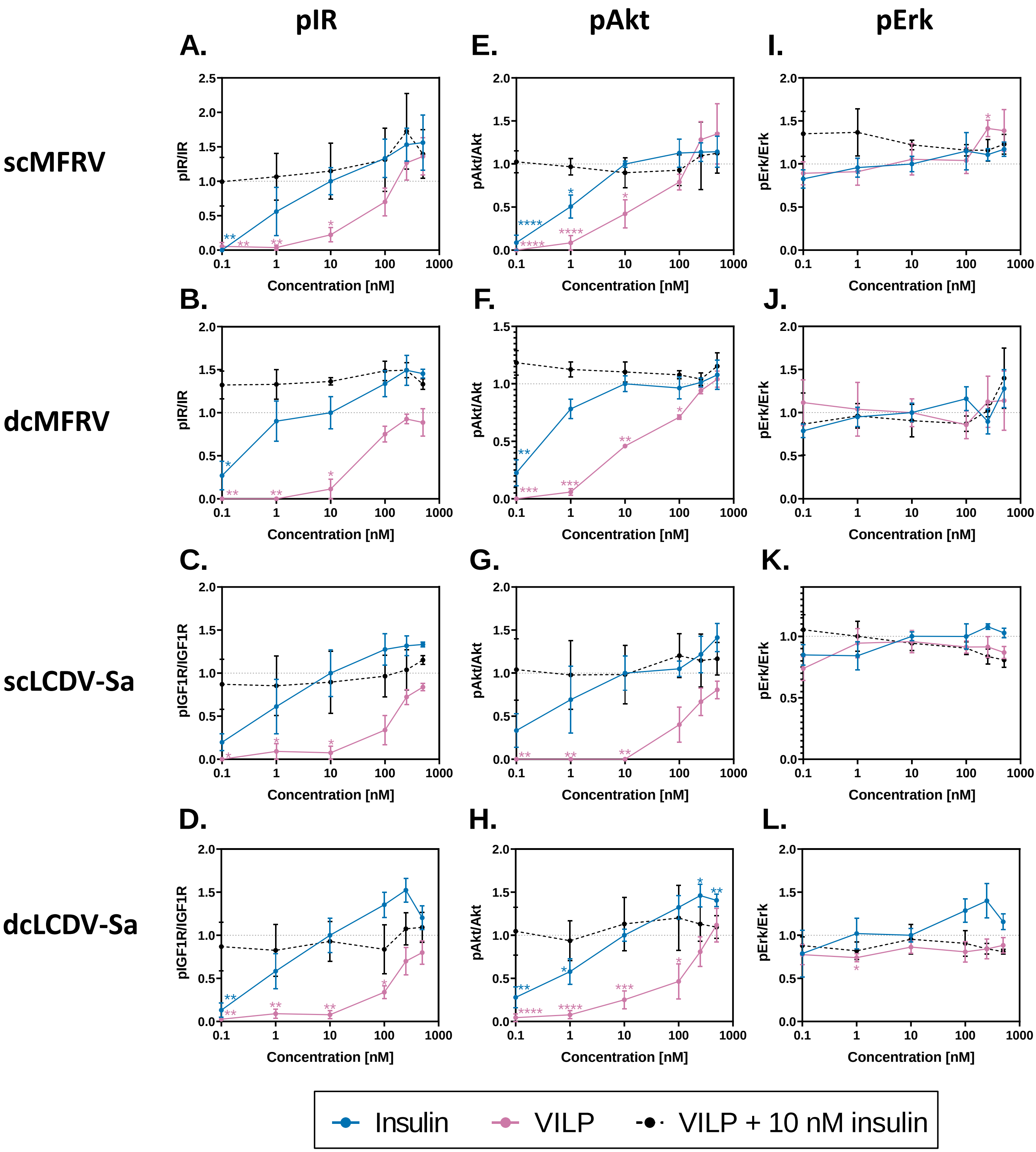

Figure S4: Western blot quantification of MFRV and LCDV-Sa-VILP stimulated insulin/IGF signaling via IR-A. R-/IR-A cells were stimulated with either human insulin, VILP or the combination of VILP and 10 nM insulin. Phosphorylation of IR-A, Akt and Erk1/2, as well as the relative amounts of the total proteins, were observed in 30 min after stimulation. Each data point is expressed as the signal of the phosphorylated version of the protein normalized to the total protein. Moreover, the data points are expressed as a fold change of 10 nM insulin, which is also indicated by a grey dashed line. Each data point represents mean ± SEM of signals quantified from at least three independent experiments. To compare each data point with 10 nM insulin, unpaired two-tailed Student's t-test was performed (*P<0.05, **<0.01, ***P<0.001, ****P<0.0001).

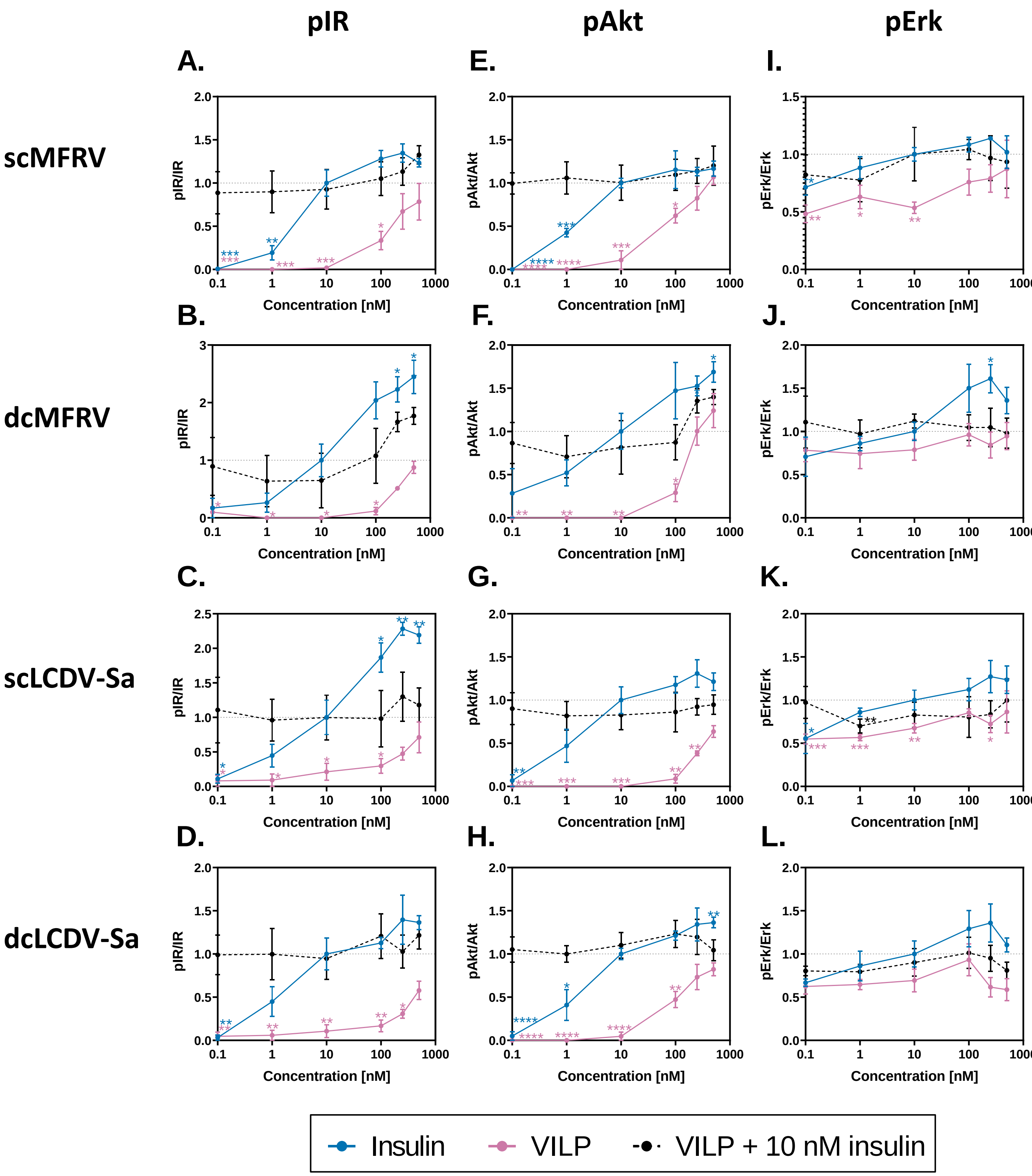

Figure S5: Western blot quantification of MFRV and LCDV-Sa-VILP stimulated insulin/IGF signaling via IR-B. R^-/IR-B cells were stimulated with either human insulin, VILP or the combination of VILP and 10 nM insulin. Phosphorylation of IR-B, Akt and Erk1/2, as well as the relative amounts of the total proteins, were observed in 30 min after stimulation. Each data point is expressed as the signal of the phosphorylated version of the protein normalized to the total protein. Moreover, the data points are expressed as a fold change of 10 nM insulin, which is also indicated by a grey dashed line. Each data point represents mean ± SEM of signals quantified from at least three independent experiments. To compare each data point with 10 nM insulin, unpaired two-tailed Student's t-test was performed (*P<0.05, **<0.01, ***P<0.001, ****P<0.0001).

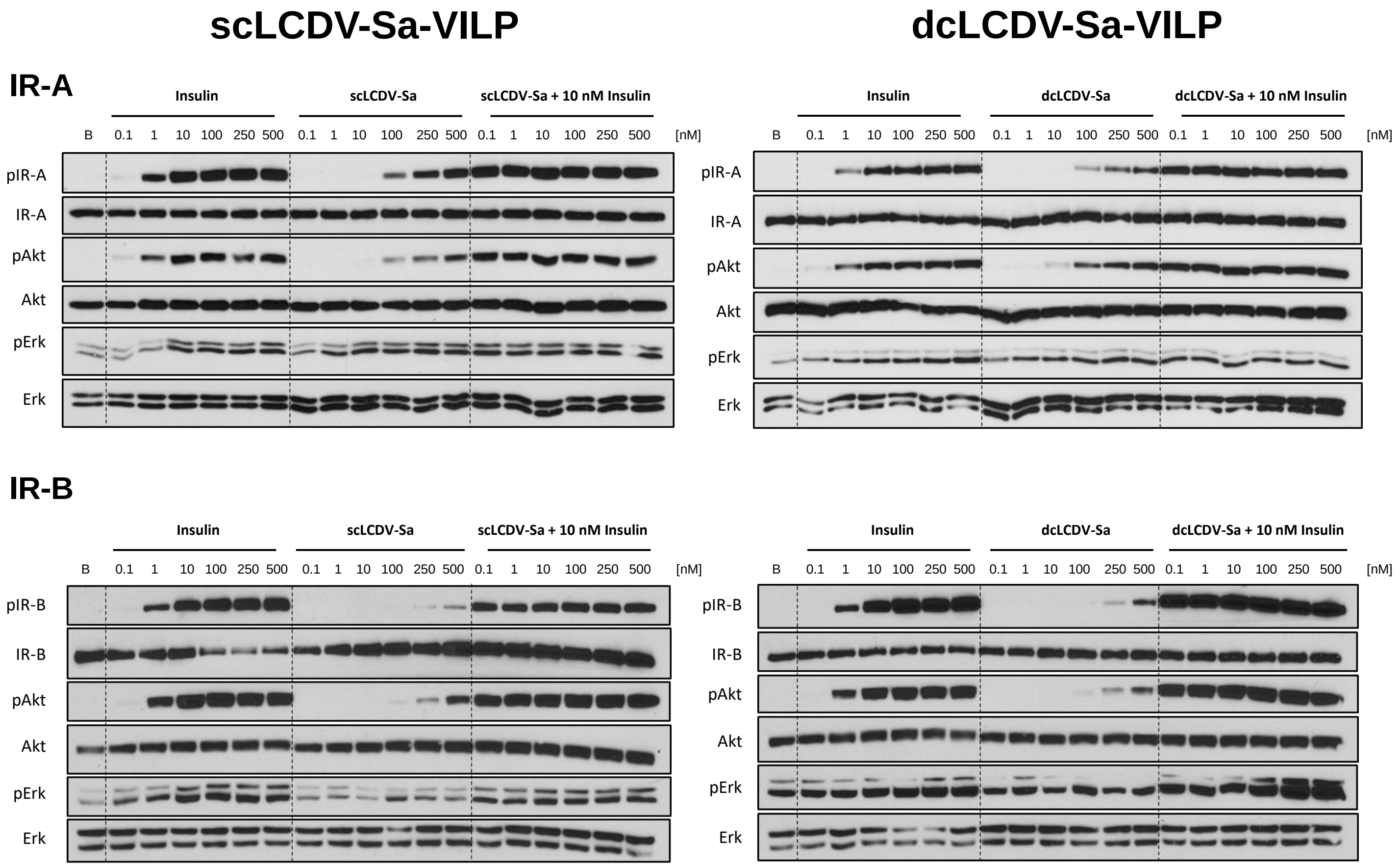

Figure S6: Representative western blots of LCDV-Sa stimulated insulin/IGF signaling on human IR-A and IR-B. R^-/IR-A or R^-/IR-B cells were used for measurements on human IR-A or IR-B, respectively. Cells were stimulated with increasing concentrations of insulin, VILP and VILP in combination with 10 nM insulin. Phosphorylation of the receptor, Akt and Erk1/2, as well as the relative amounts of the total proteins, were observed in 30 min after stimulation. Representative western blots are shown, each experiment was repeated at least three times.

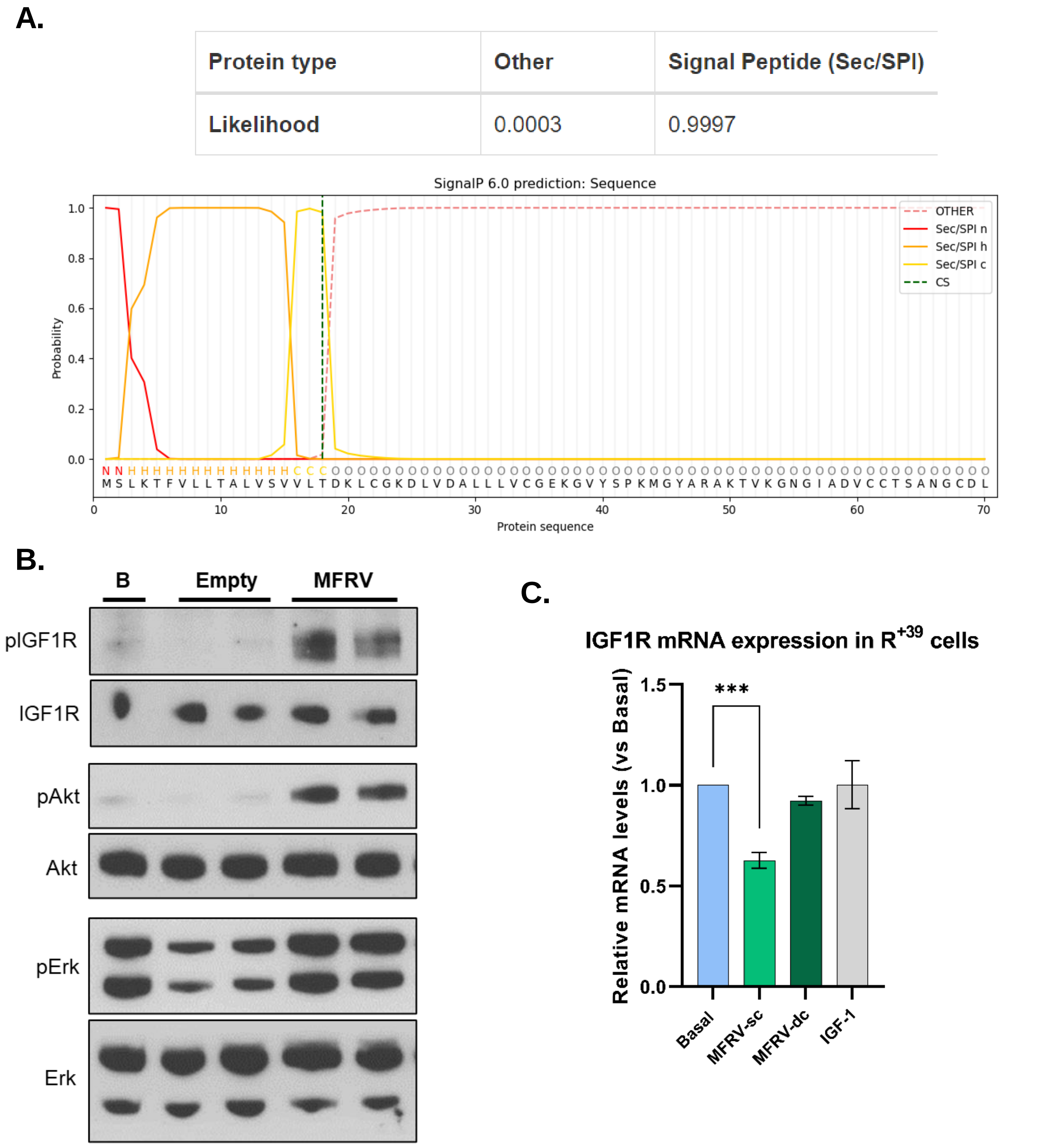

Figure S7: MFRV-VILP is are secreted following transfection and chemically synthesized scMFRV VILP decreases IGF1R gene expression. (A) SignalP 6.0 predicts that MFRV-VILP has a signal peptide with a potential cleavage site between positions 18 and 19. (**B**) Supernatants collected from AML12 cells transfected with either empty vector or MFRV-VILP were utilized to stimulate insulin/IGF signaling in serum-starved AML12 cells. This experiment confirmed the secretion of MFRV-VILP subsequent to transfection. **(C)** Stimulation of R^+ cells with scMFRV-VILP decreases IGF1R gene expression. Cells were stimulated with various ligands at 3-hour intervals for a total duration of 24 hours. Gene expression analysis was conducted after this 24-hour stimulation period. The gene expression data are normalized to β-actin levels and compared to the basal condition. Error bars represent the mean ± SEM from three independent experiments (n=3). Statistical significance was determined using an unpaired two-tailed Student's t-test (***P<0.001).

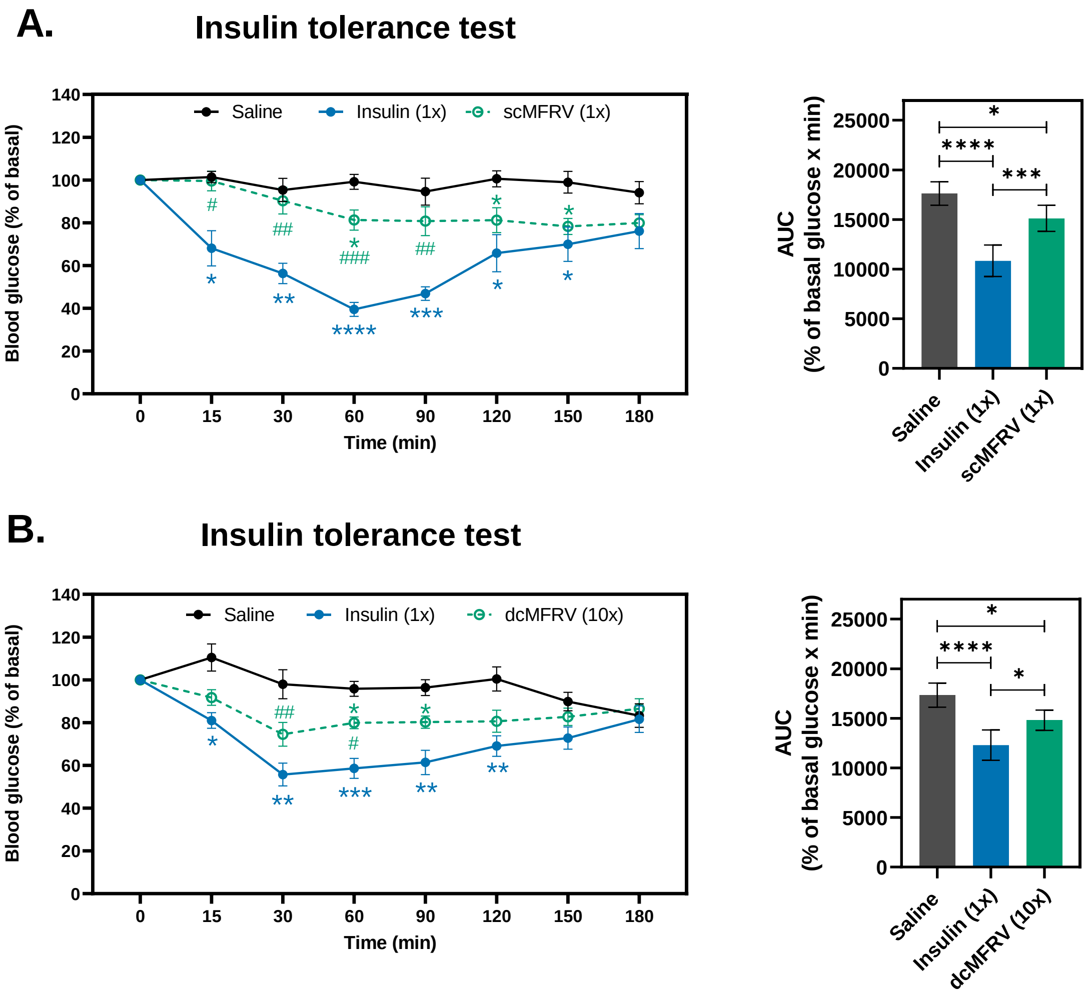

Figure S8: Insulin tolerant test. C57BL/6J mice were injected i.p. with human insulin, dcMFRV VILP, scMFRV-VILP or saline. The concentration of insulin was 6 nmol/kg in both panels, whereas the concentration of dcLCDV-Sa-VILP was 60 nmol/kg **(A)** and the concentration of scMFRV-VILP was 6 nmol/kg **(B)**. Blood glucose was measured within the range from 0 to 180 min. Data are mean ± S.E.M. Mixed-effects analysis followed by Dunnett's multiple comparisons test was applied, n 5-6 per condition. (*P < 0.05; **P < 0.01, ***P < 0.001). * is compared to saline, # is compared to human insulin. A respective chart for area under curve is shown at the right side of each panel, Ordinary one-way ANOVA followed by Tukey's multiple comparison test was applied for statistical analysis (*P<0.05; **P<0.01, ***P<0.001, ****P<0.0001).

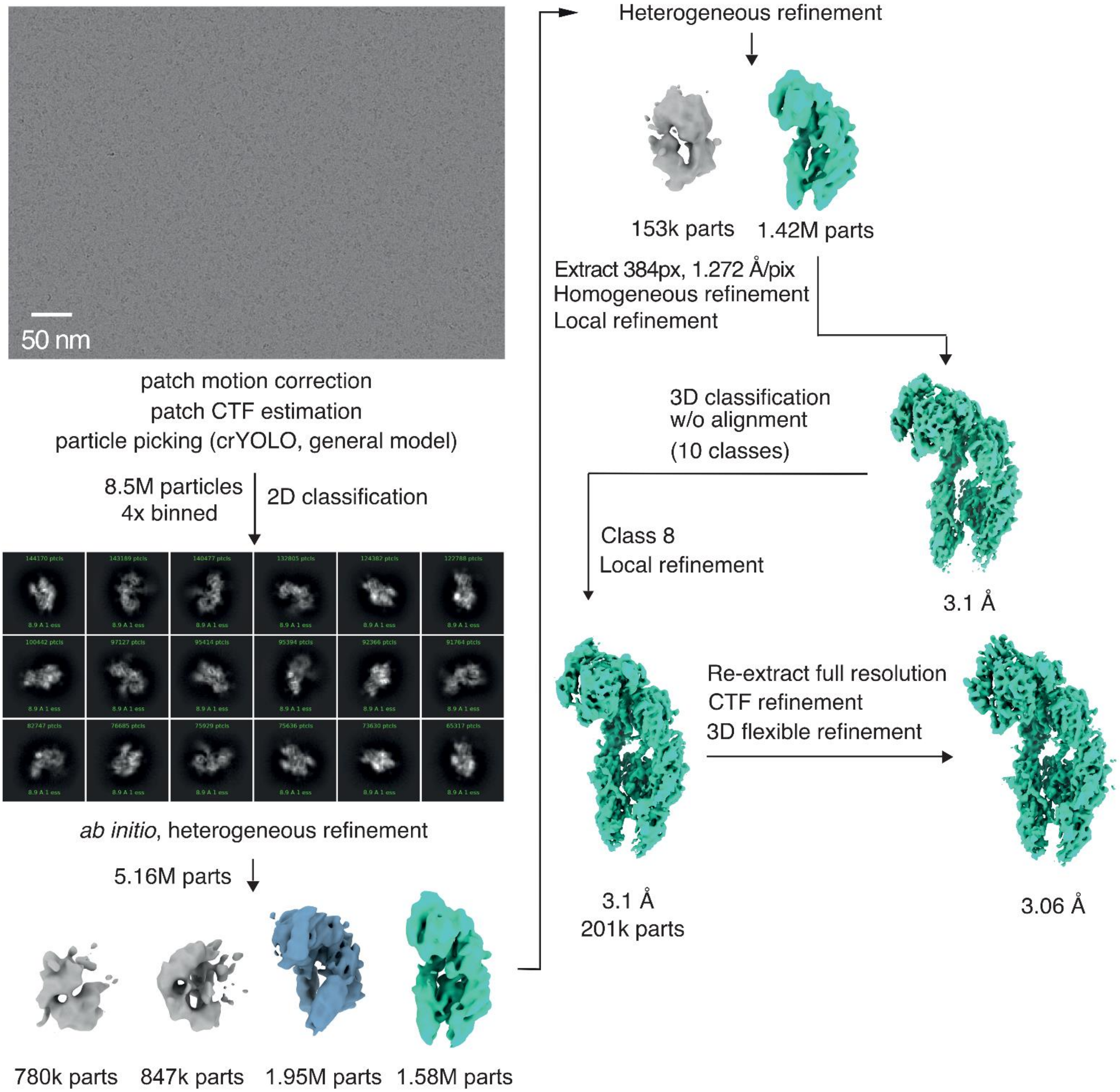

Figure S9: **Single particle analysis processing of MFRV-IGFRzip dataset**

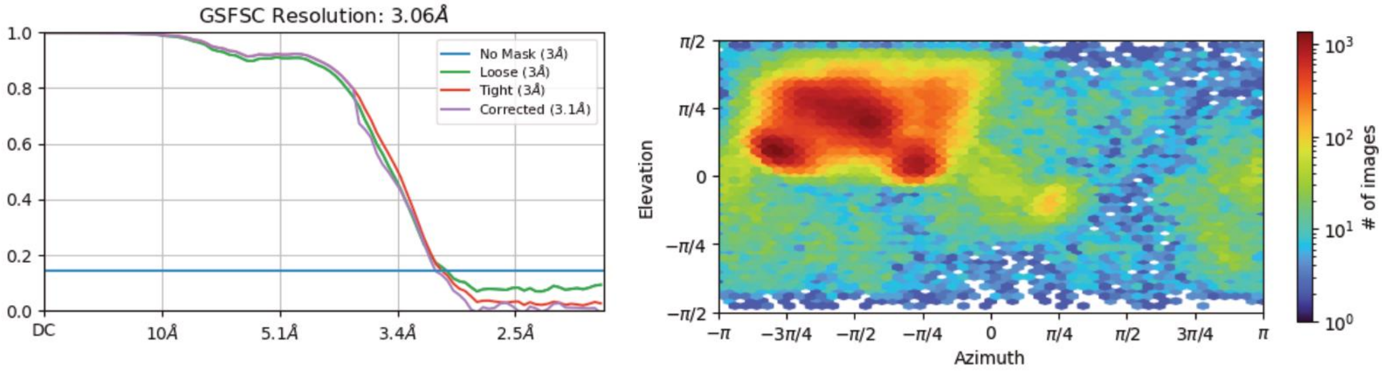

Figure S10: FSC curve and angular distribution for MFRV-IGF1Rzip

Table S1: List of primers used for amplification of ligand constructs

Ligand		Primers
**Human IGF-1**	Forward	ATT GCT AGC ATG GGA AAA A
	Reverse	AAT AAG CTT TTA AGC TGA CT
**MFRV VILP**	Forward	CAC GCT AGC ATG TCT
	Reverse	GAA AAG CTT TTA AGT TTT GCA AAA T
**LCDV-Sa VILP**	Forward	CAC GCT AGC ATG AAG G
	Reverse	GCG GTC CAC CCG TT

Table S2: List of primers used in RT-qPCR.

Protein		Primers	Reference (PMID)
		Mouse	
**IR-A and IR-B**			
	Forward	TCC TGA AGG AGC TGG AGG AGT	19406949
**IR-A**			
	Reverse	CTT TCG GGA TGG CCT GG	19406949
**IR-B**			
	Reverse	TTC GGG ATG GCC TAC TGT C	19406949
**IGF1R**			
	Forward	GGC ACA ACT ACT GCT CCA AAG AC	19406949
	Reverse	CTT TAT CAC CAC CAC ACA CTT CTG	
**β-Actin**			
	Forward	AGC CAT GTA CGT AGC CAT CCA	12490612
	Reverse	TCT CCG GAG TCC ATC ACA ATG	
		Human	
**IGF1R**			
	Forward	GGC ACA ATT ACT GCT CCA AAG AC	26862994
	Reverse	CAA GGC CCT TTC TCC CCA C	
**IGF-1**			
	Forward	ACC ATG TCC TCC TCG CAT CT	*
	Reverse	CCT GTC TCC AC ACA CGA ACT	
		Virus	
**MFRV VILP**			
	Forward	TCT GAC CGA CAA ACT GTG CG	*
	Reverse	CAC GTC CGC TAT ACC GTT CC	
**LCDV-Sa VILP**			
	Forward	TCG TTA TGT CGG TAT CGC CT	*
	Reverse	TAA ATC CCG CCG TAT TCT CCG	

* Primers were designed using NIH Primer designing tool (Primer-BLAST).

Table S3: Atomic model and cryoEM reconstruction statistics

**Model**	**MFRV-VILP-IGF1Rzip**	
PDB ID	8TAN	
Composition (#)		
Chains	3	
Atoms	13602 (Hydrogens: 0)	
Residues	Protein: 1700	
Water	0	
Ligands	0	
Bonds (RMSD)		
Length (Å) (# > 4σ)	0.002 (0)	
Angles (°) (# > 4σ)	0.697 (1)	
MolProbity score	2.15	
Clash score	5.04	
Ramachandran plot (%)		
Outliers	0.72	
Allowed	10.19	
Favored	89.09	
Rama-Z (Ramachandran plot Z-score, RMSD)		
whole (# / Z-score / RMSD)	1678 / -2.02 / 0.19	
helix (# / Z-score / RMSD)	193 / -3.05 / 0.27	
sheet (# / Z-score / RMSD)	316 / -0.83 / 0.29	
loop (# / Z-score / RMSD)	1169 / -1.22 / 0.18	
Rotamer outliers (%)	2.53	
Cβ outliers (%)	0.00	
Peptide plane (%)		
Cis proline / general	2.2 / 0.0	
Twisted proline / general	0.0 / 0.0	
CαBLAM outliers (%)	3.86	
ADP (B-factors)		
Iso / Aniso (#)	13602 / 0	
Protein (min / max / mean)	40.51 / 231.17 / 126.18	
Occupancy		
occ = 1 (%)	100.00	
**Data**		
Box		
Lengths (Å)	91.16 / 116.60 / 172.78	
Angles (°)	90.00 / 90.00 / 90.00	
Supplied Resolution (Å)	3.0	
Resolution Estimates (Å)	masked	unmasked
d FSC (half maps; 0.143)	--	
d 99 (full)	3.7	3.7
d model	3.4	3.4
d FSC model (0 / 0.143 / 0.5)	2.5 / 2.9 / 3.4	2.6 / 3.0 / 3.5
Map (min / max / mean)	-0.06 / 0.10 / 0.00	
**Model vs. Data**		
CC_{mask}	0.75	
CC_{box}	0.80	
CC_{peaks}	0.68	
CC_{volume}	0.75	

Table S4: Approximate EC_{50} and IC_{50} values from the phosphorylation assay

Stimulation with ligand of interest

Ligand	IR-A	IGF1R	
	EC_{50} [nM]	EC_{50} [nM]	IC_{50} [nM]
**Human insulin**	1.45	73.1	-
**Human IGF-1**	15.4	1.36	1546
**scMFRV**	13.9	1.19	313
**dcMFRV**	151	14.5	-
**scLCDV-Sa**	199	19.3	-
**dcLCDV-Sa**	198	282	-

Co-stimulation with ligand of interest and 5 nM IGF-1

Ligand	IGF1R
	IC_{50} [nM]
**Human IGF-1 + 5 nM IGF-1**	1484
**scMFRV + 5 nM IGF-1**	245
